# Base-resolution profiling of 5-glyceryl-methylcytosine in *Chlamydomonas reinhardtii* via deaminase-assisted sequencing

**DOI:** 10.1093/nar/gkaf955

**Published:** 2025-09-24

**Authors:** Fan-Chen Wang, Bao-Dan He, Zi-Xin Wang, Xuan Deng, Hui Chen, Wei-Ying Meng, Yu-Tao Fu, Wan-Yue Zou, Tong Ge, Yawen Li, Shu-Xia Sun, Ke-Yao Zhao, Hao-Ming Jiang, Zhi-Yan Sun, Guo-Liang Xu, Kai-Yao Huang, Jian-Huang Xue

**Affiliations:** Key Laboratory of Spine and Spinal Cord Injury Repair and Regeneration of Ministry of Education, Tongji Hospital affiliated to Tongji University, Frontier Science Center for Stem Cell Research, School of Life Sciences and Technology, Tongji University, Shanghai 200092, China; Key Laboratory of Spine and Spinal Cord Injury Repair and Regeneration of Ministry of Education, Tongji Hospital affiliated to Tongji University, Frontier Science Center for Stem Cell Research, School of Life Sciences and Technology, Tongji University, Shanghai 200092, China; Key Laboratory of Spine and Spinal Cord Injury Repair and Regeneration of Ministry of Education, Tongji Hospital affiliated to Tongji University, Frontier Science Center for Stem Cell Research, School of Life Sciences and Technology, Tongji University, Shanghai 200092, China; Key Laboratory of Algal Biology, Institute of Hydrobiology, Chinese Academy of Sciences, Wuhan 430072, China; Key Laboratory of Epigenetic Regulation and Intervention, Chinese Academy of Sciences Center for Excellence in Molecular Cell Science, Shanghai Institute of Biochemistry and Cell Biology, Chinese Academy of Sciences, University of Chinese Academy of Sciences, Shanghai 200031, China; Key Laboratory of Spine and Spinal Cord Injury Repair and Regeneration of Ministry of Education, Tongji Hospital affiliated to Tongji University, Frontier Science Center for Stem Cell Research, School of Life Sciences and Technology, Tongji University, Shanghai 200092, China; Key Laboratory of Spine and Spinal Cord Injury Repair and Regeneration of Ministry of Education, Tongji Hospital affiliated to Tongji University, Frontier Science Center for Stem Cell Research, School of Life Sciences and Technology, Tongji University, Shanghai 200092, China; Key Laboratory of Spine and Spinal Cord Injury Repair and Regeneration of Ministry of Education, Tongji Hospital affiliated to Tongji University, Frontier Science Center for Stem Cell Research, School of Life Sciences and Technology, Tongji University, Shanghai 200092, China; Key Laboratory of Epigenetic Regulation and Intervention, Chinese Academy of Sciences Center for Excellence in Molecular Cell Science, Shanghai Institute of Biochemistry and Cell Biology, Chinese Academy of Sciences, University of Chinese Academy of Sciences, Shanghai 200031, China; Key Laboratory of Epigenetic Regulation and Intervention, Chinese Academy of Sciences Center for Excellence in Molecular Cell Science, Shanghai Institute of Biochemistry and Cell Biology, Chinese Academy of Sciences, University of Chinese Academy of Sciences, Shanghai 200031, China; School of Life Science and Technology, ShanghaiTech University, Shanghai 201210, China; Key Laboratory of Spine and Spinal Cord Injury Repair and Regeneration of Ministry of Education, Tongji Hospital affiliated to Tongji University, Frontier Science Center for Stem Cell Research, School of Life Sciences and Technology, Tongji University, Shanghai 200092, China; Key Laboratory of Spine and Spinal Cord Injury Repair and Regeneration of Ministry of Education, Tongji Hospital affiliated to Tongji University, Frontier Science Center for Stem Cell Research, School of Life Sciences and Technology, Tongji University, Shanghai 200092, China; Key Laboratory of Spine and Spinal Cord Injury Repair and Regeneration of Ministry of Education, Tongji Hospital affiliated to Tongji University, Frontier Science Center for Stem Cell Research, School of Life Sciences and Technology, Tongji University, Shanghai 200092, China; Key Laboratory of Spine and Spinal Cord Injury Repair and Regeneration of Ministry of Education, Tongji Hospital affiliated to Tongji University, Frontier Science Center for Stem Cell Research, School of Life Sciences and Technology, Tongji University, Shanghai 200092, China; Key Laboratory of Epigenetic Regulation and Intervention, Chinese Academy of Sciences Center for Excellence in Molecular Cell Science, Shanghai Institute of Biochemistry and Cell Biology, Chinese Academy of Sciences, University of Chinese Academy of Sciences, Shanghai 200031, China; Key Laboratory of Algal Biology, Institute of Hydrobiology, Chinese Academy of Sciences, Wuhan 430072, China; Key Laboratory of Spine and Spinal Cord Injury Repair and Regeneration of Ministry of Education, Tongji Hospital affiliated to Tongji University, Frontier Science Center for Stem Cell Research, School of Life Sciences and Technology, Tongji University, Shanghai 200092, China

## Abstract

5gmC (5-glyceryl-methylcytosine), a vitamin C-derived hypermodified base, has been identified in the genome of *Chlamydomonas reinhardtii*. However, the global distribution of 5gmC and its role as an epigenetic mark remain poorly understood. In this study, we employed a DNA deaminase to distinguish 5gmC from 5mC (5-methylcytosine) and cytosines, enabling precise profiling of 5gmC across the genome. This deaminase-assisted sequencing demonstrates superior performance compared to the previously proposed TET-coupled bisulfite sequencing. Using both methods, we identified numerous confident 5gmC sites. Unlike 5mC, which predominantly occurs at CpG sites, 5gmC is preferentially located in CHH contexts. Remarkably, over half of 5gmC sites are mutually exclusive from 5mC, with the remaining sites inversely correlated with 5mC levels, suggesting a role in active DNA demethylation. Additionally, 5gmC is enriched within introns, contrasting with the more extensive localization of 5mC in intergenic and promoter regions. Importantly, 5gmC levels are positively correlated with transcription, while 5mC typically exhibits an inverse relationship with gene expression, consistent with the enrichment of 5mC but lack of enrichment of 5gmC at H3K9me1-marked repressive chromatin. Collectively, these findings suggest that 5gmC is not only an intermediate for active DNA demethylation but also functions as a stable epigenetic mark, potentially influencing transcriptional regulation independently of 5mC.

## Graphical Abstract

**Graphical Abstract:**
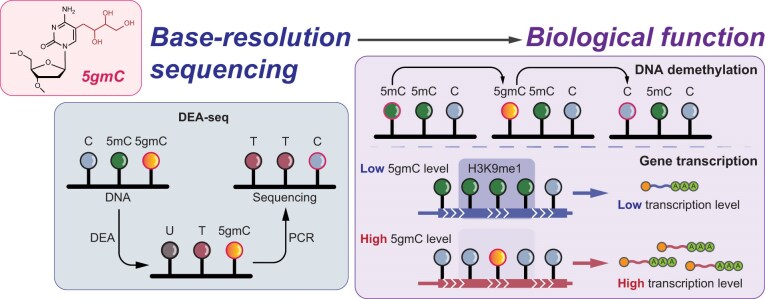

## Introduction

DNA modifications are key components of epigenetic marks, which play crucial roles in transcriptional regulation and various biological processes. Recently, more than 10 types of DNA modifications have been identified in eukaryotes [1]. Among these, DNA methylation is the most prevalent modification across nearly all kingdoms [2]. In mammalian cells, DNA methylation primarily occurs at the C5 position of cytosines (C), forming 5-methylcytosine (5mC), which is catalyzed by highly conserved C5-DNA methyltransferases (DNMTs). 5mC predominantly occurs in CpG dinucleotide contexts. Over 60% of CpG sites are methylated in the genome, while more than 98% of non-CpG sites remain unmethylated [3, 4]. In mammals, *de novo* DNA methylation is mediated by both DNMT3A and DNMT3B. During DNA replication, hemi-methylated, symmetrical CpG sites recruit DNMT1 via UHRF1 to maintain DNA methylation [5, 6]. 5mC is generally associated with gene silencing, particularly when located in the enhancer or promoter regions of protein-coding genes or within repetitive elements. However, 5mC in the gene body may enhance gene expression by preventing alternative transcription [7]. In plants and other lower eukaryotes, 5mC is found in both CpG and non-CpG contexts, contributing to more complex regulatory roles in gene expression [8]. In addition to 5mC, other modified bases such as *N*4-methylcytosine (4mC) and *N6*-methyladenosine (6mA) have also been discovered in eukaryotes [9–11]. However, the distribution and associated methyltransferases for these modifications remain to be further explored [1, 12].

Apart from DNA methyltransferases, dioxygenases represent another family of proteins with specific roles in DNA modification. The first DNA dioxygenase identified was involved in the synthesis of base J (β-D-glucosyl-hydroxymethyluridine), which was initially discovered in kinetoplastids [13]. Base J is synthesized through two successive steps. The first step involves the oxidation of thymine to 5-hydroxymethyluridine (5hmU), catalyzed by JBP1 and JBP2. The 5hmU is then further modified by a glucosyltransferase to form base J. JBP1 and JBP2 belong to a dioxygenase superfamily that is dependent on α-ketoglutarate and Fe(II) [14]. Conserved with the catalytic domain of JBP1/2, three paralogous human proteins including TET1, TET2, and TET3 were identified to exhibit oxidation activity on 5mC, generating 5-hydroxymethylcytosine (5hmC), 5-formylcytosine (5fC), and 5-carboxylcytosine (5caC) in the genome. The latter two modified bases can be excised by thymine DNA glycosylase (TDG), initiating the base excision repair (BER) pathway, which results in the replacement of modified cytosines with unmodified ones [15–17]. Thus, TET-mediated DNA oxidation is considered an intermediate process in active DNA demethylation in mammals. Furthermore, oxidation of 5mC sequesters UHRF1, which is required for maintenance DNA methylation, leading to passive DNA demethylation during DNA replication [18].

TET/JBP homologues have been predicted to be present in a wide range of eukaryotes, including *Chlamydomonas reinhardtii* (*C. reinhardtii*) [19]. Interestingly, these homologues appear to exhibit distinct enzymatic activities toward various substrates [1]. In *C. reinhardtii*, a TET homologue named CMD1 (5mC modifying enzyme 1) catalyzes the conversion of 5mC into 5-glyceryl-methylcytosine (5gmC) using vitamin C, rather than α-ketoglutarate, as the co-substrate [20, 21]. 5gmC has also been shown to function as an intermediate in 5mC demethylation and play a critical role in the feedback regulation of photosynthesis [20]. However, the genomic distribution of 5gmC remains unknown, and whether 5gmC acts as an independent epigenetic mark that can influence gene expression is still under investigation.

To reveal the roles of 5gmC, accurately determining its genome-wide distribution is essential. Since modifications on these nucleotides do not alter the base-pairing properties, distinguishing modified nucleotides from unmodified ones can be challenging. A variety of methods have been developed to read DNA epigenetic modifications. Bisulfite sequencing (BS-seq), the gold standard for detecting 5mC at base resolution, converts unmodified cytosines into uracils (U), which are read as thymines (T) after polymerase chain reaction (PCR), while 5mC remains unchanged and read as C [22]. Numerous methods derived from BS-seq have been developed to detect 5hmC, 5fC, and 5caC [23]. For example, TET-assisted bisulfite sequencing (TAB-seq) has been introduced to directly detect 5hmC. In this method, TET proteins oxidize 5mC to 5caC, which is read as T after conventional BS-seq, while 5hmC is protected from further oxidation by glucosylation into glucosylated 5hmC (5ghmC) and thus remains unchanged [24]. However, BS-seq will damage DNA strands and reduce sequencing quality. Additionally, the conversion rate of 5mC into 5caC is limited by the activity of TET proteins, which could lead to false-positive results in TAB-seq. Another method, APOBEC-coupled epigenetic sequencing (ACE-seq), relies on enzyme-mediated C-to-T conversion to distinguish 5hmC from 5mC. APOBEC deaminase converts both C and 5mC into U or T, while 5hmC remains unaffected due to its glucosylation [25]. These studies highlight the sequence bias of 5hmC, suggesting distinct mechanisms for its maintenance and its roles in gene transcription [26].

Similarly, a TET-coupled bisulfite sequencing (TET-BS-seq) was developed in our previous study to distinguish 5gmC from 5mC [20], as 5gmC resists both TET-mediated DNA oxidation and bisulfite-mediated C-to-T conversion without further modification. However, genome-wide profiling of 5gmC in *C. reinhardtii* is still lacking. Furthermore, since the level of 5gmC is much lower than that of 5mC or unmodified C in the genome [27], accurate determination of 5gmC may be hindered by the insufficient conversion rate of 5mC or C through TET-BS-seq. In this study, we introduce an alternative method aimed at simplifying the procedure and improving the conversion rate of 5mC and C, facilitating the identification of confident 5gmC sites. Specifically, we utilized a commercial DNA deaminase mix (DEA, see Material and Methods) to develop the deaminase-assisted sequencing (DEA-seq) for the identification of 5gmC. DEA efficiently deaminates both C and 5mC, converting them into U or T, while leaving 5gmC unaffected. The selective resistance of 5gmC to deamination by DEA mirrors the resistance of 5ghmC to APOBEC-mediated deamination in ACE-seq [25], making DEA a useful tool for differentiating 5gmC from other cytosines. Thus, this single-step treatment is straightforward and enables efficient profiling of 5gmC across the entire genome of *C. reinhardtii*.

5gmC was found to be enriched in CHH contexts in the genome of *C. reinhardtii* according to DEA-seq analysis. More than half of the 5gmC sites are located within introns, and the highest modification levels were observed in intronic and distal intergenic regions. In contrast, 5mC is predominantly found at CpG and CHH sites, with the highest modification levels occurring in intergenic regions based on the whole genome bisulfite sequencing (WGBS) data. 5gmC sites are mutually exclusive with 5mC or show an inverse correlation with 5mC levels, supporting the role of 5gmC in promoting DNA demethylation [20]. However, 5gmC is also stably present in intronic regions of specific genes, suggesting that 5gmC may serve as a stable epigenetic mark. Additionally, 5gmC is positively correlated with gene transcription, particularly when located in the upstream regions of gene bodies. This is consistent with that 5gmC is less enriched at H3K9me1 sites, which is a repressive mark colocalized with 5mC in *C. reinhardtii* [28]. Overall, this study introduces a DEA-seq method for 5gmC profiling and reveals the genome-wide distribution of both 5gmC and 5mC in *C. reinhardtii*. Our findings suggest that 5gmC exhibits a distinct distribution pattern and may have a direct impact on gene transcription independent of 5mC. This work also provides a valuable resource for future investigations into DNA modifications in green algae and potentially other organisms.

## Material and methods

### C. reinhardtii strains and culture conditions

The *C. reinhardtii* strains (Wild-type CC125 and CC5325, and the *Dnmt1* knockout strain) were cultured mixotrophically in TAP medium on a rotary shaker at 25°C, with a light intensity of 20 μmol photons m^−2^•s^−1^.

### Genomic DNA and RNA isolation from *C. reinhardtii*

Total DNA was isolated using the CTAB method as described previously [20] and dissolved in nuclease-free water for further analysis. Total RNA was extracted using Trizol (Sangon) according to the manufacturer's instructions.

### Methylation of lambda DNA by M.SssI

For each reaction, 1 μg of lambda DNA (λDNA, dam-, dcm-; Takara) was incubated with 1 μl of M.SssI methyltransferase (NEB) in the presence of 1 × reaction buffer and 160 μM S-adenosylmethionine (SAM), following the manufacturer's protocol. After the reaction, the DNA was treated with proteinase K (NEB) to remove proteins and subsequently purified using the DNA Clean & Concentrator-5 Kit (Zymo Research), according to the manufacturer's instructions.

### PCR amplification of 5mC-containing spike-in DNA

The spike-in 5mC-DNA fragment used in next-generation sequencing was prepared by PCR using 5m-dCTP (5-methyl-dCTP) in place of dCTP. The PCR was performed with the following primers: Forward primer: GAATTCTTGCAGCACTAGTGCATC; and Reverse primer: CTTTGCAACTTTTAAATCAC. The template was a random synthetic 480 bp fragment. The DNA sequence is as follows: 5′-GAATTCTTGCAGCACTAGTGCATCTATAAGTTATCTCAAATCAAGAAATCAGTCTAATGAGAATTTCAATAACTTCAGCAATTTAAGCTGCATGCATCAGTGTCATCGTTATTTTTTTTTTGAGACGTAGTCATGCTCTGTTGCTGAGTCTGCAGTACAGTGACGAGATATCGACTCAGCACAACATCTGCATCACATGTTCAAGCGATTCTCATGCTTCAGCTTGCAGAGTAGCTGTCACTACAGACACTGAGCAGCATGCGTGACTAATTTTTGTATTTTTAGTAGAGAGTGCATTTCGTCATGTTGTACAGTCTAGTTTCAAACTCATGACTTCAGTTGATCTAACTGACACGATCTCAGAATTTACTGTCATTACAGTACTGTCACACAGTGACAGTCATTTTTCTTAATTTTTAAAAATATTAAAGTTTTATCTCATTCGTGTTGAAGCATATTCGTGATTTAAAAGTTGCAAAG-3′. The PCR reaction was performed using TaKaRa Taq™ Hot Start Version (TaKaRa) and the product was purified with SanPrep Column DNA Gel Extraction Kit (Sangon) according to the manufacturer's instructions.

### CMD1 recombinant protein expression and purification

The pPEI-His-SUMO-CMD1 plasmid was generated as described previously [20] and transformed into E. coli strain BL21 (DE3). The bacterial cells were grown to an optical density (OD600) of 0.6 and induced with 0.5 mM isopropyl-β-D-thiogalactopyranoside (IPTG) at 16°C for 16 h. After lysis with lysis buffer (1 × PBS, 2 mM Imidazole, 1 M NaCl), the His-tagged CMD1 protein was bound to Ni-NTA beads (Beyotime) and cleaved from the His-SUMO tag by overnight incubation with His-tagged Ulp1 protease at 4°C. The protein was then eluted with storage buffer (50 mM HEPES, pH 7.0, 50 mM NaCl, 10% glycerol) and concentrated to 2 μg/μl using Nanosep-30K centrifugal filters (Pall).

### CMD1 reaction assay

For each reaction, 500 ng of methylated lambda DNA was incubated with 10 μg of CMD1 protein in a total volume of 100 μl at 37°C for 2 h. The reaction was carried out in the presence of 50 mM HEPES (pH 7.0), 50 mM NaCl, 3 mM L-ascorbic acid and 0.2 mM Fe(NH_4_)_2_(SO_4_)_2_. After treatment with proteinase K (NEB), the DNA was purified using the DNA Clean & Concentrator-5 Kit (Zymo Research), following the manufacturer's instructions.

### 5mC and 5gmC quantification by UPLC-MS/MS analysis

To quantitatively determine the content of 5mC and 5gmC nucleosides, genomic DNA or CMD1-treated DNA was first digested with nuclease P1 (NEB) at 37°C for at least 2 hours, followed by dephosphorylation with calf intestinal alkaline phosphatase (CIAP, Takara) at 37°C for an additional 1 h. The samples were then centrifuged, and the supernatants were collected for analysis. The DNA was subjected to multiple reaction monitoring (MRM)-based UPLC-MS/MS analysis. MS analysis was performed using a UPLC system (ACQUITY UPLC I-Class, Waters) coupled to a triple quadrupole mass spectrometer (Triple Quad^TM^ 6500 + LC-MS/MS, SCIEX). Separation was achieved using an ACQUITY^TM^ Premier HSS T3 Column (100 Å, 1.8 μm, 2.1 mm × 100 mm, Waters). The mobile phases used for compound separation were: A, 0.1% formic acid in pure water; and B, 100% methanol. The linear gradient elution profile was as follows: 0% B (0–1 min), 0–10% B (1–4 min), 10–50% B (4–6 min), 50–95% B (6–6.5 min), hold at 95% B for 1 min, 95–0% B (7.5–7.6 min), followed by equilibration at 0% B until 10 min. The flow rate was set to 0.3 ml/min. Optimized MRM transition parameters for each nucleoside were obtained using pure compound standards. The quantifier transitions for each nucleoside were: 5mC: 242.1/126.1 (CE 17, DP 20); dC: 228.1/112.1 (CE 20, DP 20); dG: 268.1/152.1 (CE 20, DP 60); 5gmC: 332.1/216.1 (CE 20, DP 15). All compounds were measured in positive ESI mode. Retention times for each compound were determined by measuring the corresponding MRM transitions on the HSS T3 column: For 5mC: 3.15 min; dC: 2.31 min; dG: 4.50 min; 5gmC: 2.71 min. The amount of each nucleoside was quantified by interpolating the peak areas of the quantifier MRM transitions from the standard curves.

### WGBS and TET-BS-seq

For TET-BS-seq, genomic DNA was firstly sonicated into approximately 600 bp fragments using a Covaris M220. The sonicated DNA (with ∼0.1% lambda DNA and 5mC-DNA fragments as a spike-in control) was incubated with recombinant TET proteins (Vazyme) for oxidation following the manufacturer's instructions. Briefly, 200 ng of DNA was incubated with 10 μl of TET proteins in a total volume of 20 μl at 37°C for 3 h in the presence of the appropriate oxidation buffer. After oxidation, the TET-treated or untreated DNA (for WGBS) was directly subjected to bisulfite treatment according to the manufacturer's instructions (Zymo Research).

The purified bisulfite-treated DNA was then used for sequencing library preparation with the EpiArt DNA Methylation Library Kit for Illumina V3 (Vazyme, NE103-01), following the manufacturer's protocol. Briefly, DNA libraries were generated by 3′ adapter ligation, extension, 5′ adapter ligation and library amplification. Then the libraries were analyzed using an Agilent 2100 Bioanalyzer to assess their quality and sequenced on an Illumina NovaSeq X Plus platform by 150 bp paired-end sequencing.

### DEA-seq

For DEA-seq, genomic DNA was firstly sonicated into approximately 300 bp fragments using a Covaris M220. A small fraction of the 5mC-DNA fragments and unmodified lambda DNA (∼0.1%) was added as a spike-in control. Then, 16 μl (20 ng) of DNA dissolved in nuclease-free water was mixed with 4 μl of 0.1 M NaOH and heated at 50°C for 10 min to denature the DNA. After fast-cooling, the DNA was treated with 1 μl of DEA enzyme mix (Vazyme, EM301-C110) in the presence of 10 μl of DEA reaction buffer and 1 μl of BSA, and incubated at 37°C for 3 h. The treated DNA was purified using DNA Clean & Concentrator-5 Kit (Zymo research) according to the manufacturer's instructions and then subject to sequencing library construction as done for WGBS. Notably, DEA enzyme mix is a component of the EpiArt DNA Enzymatic Methylation Kit (Vazyme, Catalog number: EM301; https://bio.vazyme.com/products_7/154.html).

### RNA sequencing

First, rRNA was depleted from total RNA using the Ribo-off rRNA Depletion Kit according to the manufacturer's instructions (Vazyme). RNA library construction was then performed using the VAHTS Universal V10 RNA-seq Library Prep Kit for Illumina (Vazyme, NR606-01), following the manufacturer's instructions. The libraries were assessed using the Agilent 2100 Bioanalyzer and sequenced on the Illumina platform using 150 bp paired-end sequencing.

### Data analysis

Sequencing data from WGBS were processed using Trimmomatic (v0.39) [29] for trimming and filtering, followed by FastQC (v0.12.1, https://www.bioinformatics.babraham.ac.uk/projects/fastqc/) for quality control. Clean reads were aligned to the reference genome (*C. reinhardtii* CC_4532_707_v6.0, downloaded from https://phytozome-next.jgi.doe.gov/) and to the spike-in sequence (used to determine non-conversion rate) using Bismark (v0.24.2) [30]. After deduplication and methylation extraction via deduplicate_bismark and bismark_methylation_extractor (both Bismark sub-tools), the following thresholds were applied to filter 5gmC sites: counts of unconverted cytosines > 10 and ratio > 17%. These thresholds were determined through gradient testing to ensure appropriate 5gmC abundance and maximum overlap between DEA-seq and TET-BS-seq methods.

RNA-seq data were processed similarly to WGBS data, with trimming, filtering, and quality control performed using Trimmomatic and FastQC. Clean reads were aligned to the *C. reinhardtii* CC_4532_707_v6.0 reference using Hisat2 (v2.2.1) [31]. Transcript assembly and expression quantification were then performed with StringTie (v2.2.3) [32]. For site filtering, methylation distribution analysis, transcriptional regulation, and chart plotting, the following Python modules and R packages were used: Python: Pandas (v2.2.2) [33], NumPy (v1.26.4) [34], Matplotlib (v3.8.4) [35], and SciPy (v1.13.1) [36]; R: ChIPseeker (v1.40.0) [37], GenomicFeatures (v1.56.0) [38], and ggplot2 (v3.5.1) [39].

For profiling 5mC and 5gmC modification around transcripts, deepTools (v3.5.5) [40] was used for data processing and visualization. Gene Ontology (GO) and Kyoto Encyclopedia of Genes and Genomes (KEGG) pathway analyses were performed using the STRING website (https://cn.string-db.org) with default settings.

For the 5mC/5gmC-TE overlapping analysis, gene transcripts and TE information were extracted from the corresponding genome annotation files (*C. reinhardtii* CC_4532_v6.1 and CC_4532_707_v6.1.repeatmasked_assembly_v6.0), downloaded from the Phytozome database. For the 5mC/5gmC-histone methylation analysis, chromatin immunoprecipitation sequencing (ChIP-seq) data for histone modifications (H3K9me1, H3K4me3, and H3K9me3) were downloaded from GEO (GSE245611). For the comparison of genes containing 5gmC and genes regulated by CMD1, raw data of RNA-seq (WT and *CMD1* KO) were downloaded from SRA (PRJNA506067) and processed by the same method as described above.

All analysis scripts used in the study are available upon request.

## Results

### The establishment of DEA-seq for 5gmC sequencing

**Figure 1.**
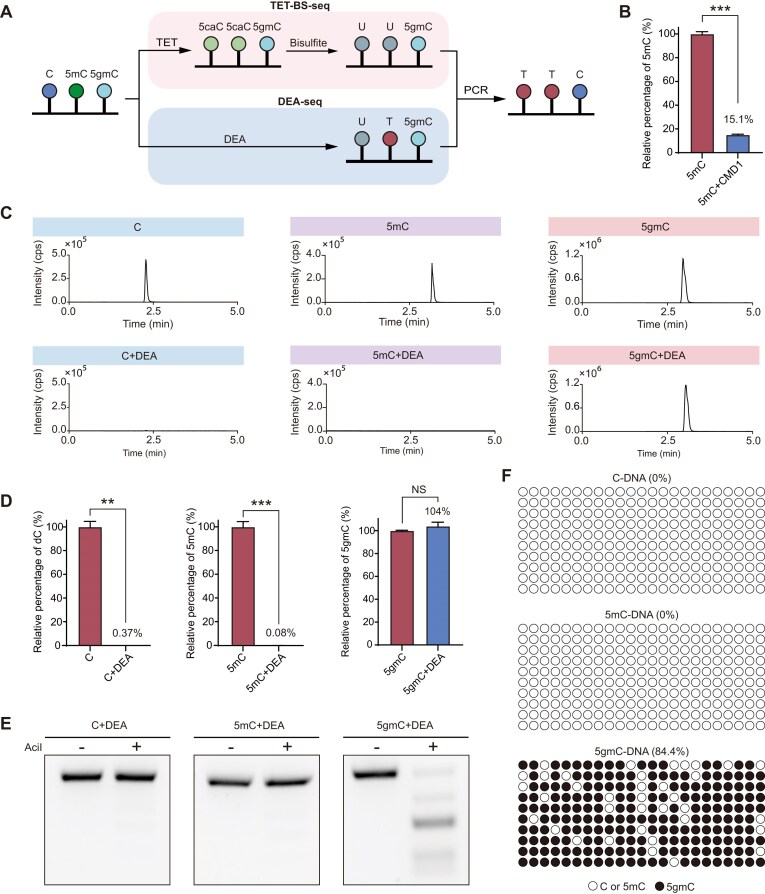
Establishment of DEA sequencing. (**A**) Schematic overview comparing DEA-seq and TET-BS-seq methodologies for distinguishing 5gmC from 5mC and C. (**B**) Relative abundance of 5mC in methylated lambda DNA after treatment with CMD1. Data are mean ± SD from two independent biological replicates. (**C**) Representative images showing MS analysis of C, 5mC, and 5gmC in DNA after DEA treatment. (**D**) Quantitative analysis of the differently modified nucleosides after DEA treatment. Data are mean ± SD from two independent biological replicates. (**E**) Restriction enzyme digestion analysis of differently modified DNA after DEA treatment and PCR amplification. (**F**) Sanger sequencing analysis of lambda DNA containing different modified cytosines after DEA treatment. Each circle represents a CpG site in the lambda DNA sequence.

In our previous work, we developed TET-BS-seq to distinguish 5gmC from 5mC (Fig. 1A) [20]. To validate this approach, we prepared M.SssI-treated lambda DNA, which is used for the CMD1-mediated generation of 5gmC-containing DNA. CMD1 proteins were purified from E. coli and the enzymatic activity was confirmed by mass spectrometry (MS) analysis (Supplementary Fig. S1A–C). Both 5mC-DNA and 5gmC-DNA were subjected to TET-BS-seq. As anticipated, most of the 5mC was converted into T after TET-mediated oxidation and bisulfite treatment, followed by PCR amplification, while 5gmC remained unaltered, resulting in an intact restriction site in the 5gmC-DNA (Supplementary Fig. S1D). Sanger sequencing further confirmed the effectiveness of TET-BS-seq to distinguish 5gmC from C and 5mC (Supplementary Fig. S1E). However, the two-step conversion process left approximately 3% of the 5mC unchanged, reading as C, which could introduce potential false positives in 5gmC profiling.

To improve the conversion efficiency of 5mC and accurately identify 5gmC loci across the *C. reinhardtii* genome, we propose a one-step approach, DEA-seq, for directly detecting 5gmC (Fig. 1A). This method was inspired by the discovery that the single-strand DNA deaminase APOBEC3A can efficiently deaminate both C and 5mC [41]. We hypothesized that the bulky glyceryl group on 5gmC may hinder the deaminase activity, thus allowing 5gmC to be distinguished from C and 5mC (Fig. 1A). In DEA-seq, we utilized a commercial DNA deaminase mix (DEA) instead of recombinant APOBEC3A to test its deaminase activity against C, 5mC and 5gmC in lambda DNA. 5gmC-DNA was prepared by CMD1 reaction (Fig. 1B). As expected, DEA completely deaminated both C and 5mC, while almost all of the 5gmC remained unchanged, as confirmed by MS analysis (Fig. 1C, D). Additionally, restriction enzyme digestion and sequencing analysis yielded similar results (Figs 1E, F), further validating that 5gmC effectively blocks the deaminase activity of DEA, thus can be directly read out through sequencing. Notably, DEA exhibited comparable activity in 5mC deamination comparing to C (Fig. 1D), with no residual 5mC left after DEA treatment (Fig. 1F), indicating that incomplete deamination of 5mC has minimal impact on 5gmC detection by DEA-seq, thereby achieving a lower false-positive rate in comparison with TET-BS-seq. Although DEA was also sensitive to 5fC, 5caC, and 5ghmC, none of these DNA modifications were detected in the genome of *C. reinhardtii* (Supplementary Fig. S1F, G). Additionally, DEA exhibited no preference for different genomic contexts (CpG, CHG or CHH, where H represents A, C, T) during the deamination process, using either C- or 5mC-DNA as substrates (Supplementary Fig. S2A–D). Therefore, the DEA-seq approach offers an efficient and direct method for the genome-wide detection of 5gmC.

### Whole genome profiling of 5gmC in *C. reinhardtii*

To assess the genome-wide distribution of 5gmC in *C. reinhardtii*, we applied both DEA-seq and TET-BS-seq to identify confident 5gmC sites in the genome of two commonly used *C. reinhardtii strains*, CC125 (mating type +) and CC5325 (mating type −) [42]. A PCR-amplified 5mC-DNA fragment and unmodified lambda DNA were spiked into the genomic DNA to evaluate the C to T conversion rate of both C and 5mC. To further eliminate potential false-positive 5gmC sites, we introduced a *Dnmt1*-knockout (KO) strain, in which 5mC levels are reduced to less than 10% of those observed in wild-type (WT) strains (Supplementary Fig. S3A). Notably, no 5gmC was detected in the *Dnmt1* KO strain by MS analysis, providing an effective negative control (Supplementary Fig. S3B).

The sequencing data were analyzed using standard pipelines (Supplementary Fig. S4A). After trimming and filtering, the sequenced reads were mapped to the *C. reinhardtii* genome and de-duplicated. An average coverage of 91% and a depth of 95–145× were generated (Supplementary Fig. S4B). The uniquely aligned reads were extracted for methylation analysis. To reduce background noise and potential false-positive signals, 5gmC sites were further filtered by subtracting signals detected in the *Dnmt1* KO strain. Since DEA-seq does not involve bisulfite treatment, which can cause significant DNA degradation [43], the overall quality of the sequencing data from DEA-seq was comparable with that of TET-BS-seq (Supplementary Fig. S4B-D), although fewer DNA was used for sequencing.

Accordingly, DEA-seq showed higher conversion rates (>99.7%) for both 5mC and unmodified C across different genomic contexts in the spike-in DNA compared to TET-BS-seq (Supplementary Fig. S5A). Notably, whereas TET-BS-seq displayed context-dependent variation in conversion efficiency for 5mC, the DEA enzyme demonstrated consistent and robust deamination activity toward 5mC and C across diverse genomic contexts (Supplementary Fig. S5B, C), highlighting the advantage of DEA-seq for 5gmC sequencing. Therefore, the primary analysis focused on DEA-seq data, though TET-BS-seq analyses were also used for validation.

As approximately 0.2% of 5mC and unmodified C remained unaltered after DEA-seq (Supplementary Fig. S5), it is crucial to establish an appropriate cut-off for identifying confident 5gmC sites. We tested different depth and ratio thresholds as potential cut-offs (Supplementary Fig. S6A). The resulting 5gmC abundance was calculated, and overlapping sites identified by both DEA-seq and TET-BS-seq were determined. Based on this, we selected a count of 10× for unconverted cytosines (5gmC) and a modification ratio of 17% as the final cut-off for both DEA-seq and TET-BS-seq analysis. These thresholds yielded a 5gmC abundance similar to previously reported data, accounting for approximately 10 ppm of total cytosines (Supplementary Fig. S6B) [27]. Furthermore, more overlapping sites were identified from both sequencing methods with these cut-offs (Supplementary Fig. S6C). Since WGBS cannot distinguish 5mC from 5gmC, confident 5mC sites were identified by subtracting the ratio of 5gmC from WGBS data [24, 25], denoted as BSΔDEA or BSΔTET-BS, respectively.

### Distribution of 5gmC at CpG and non-CpG sites

Using this analysis pipeline, we identified numerous confident 5mC and 5gmC sites in the *C. reinhardtii* genome. Principal component analysis (PCA) demonstrated good consistency between DEA-seq and TET-BS-seq for 5gmC detection in wild-type CC125 and CC5325 strains (Supplementary Fig. S7A). In contrast, the two methods showed poor concordance in *Dnmt1* knockout cells, suggesting that background noise or nonspecific signals may contribute to false-positive 5gmC calls in the absence of genuine 5gmC modification (Supplementary Fig. S7A).

**Figure 2.**
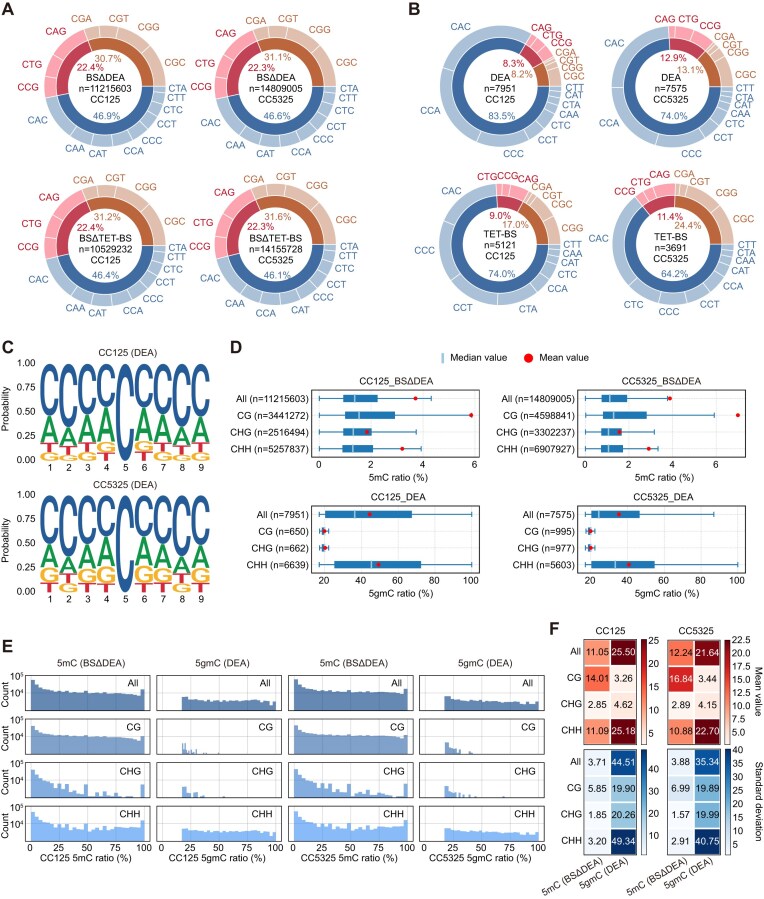
Distribution of 5gmC in different C-contexts of the genome. (**A, B**) Site-specific distribution of 5mC (**A**) and 5gmC (**B**) across different C-contexts (CG, CHG, and CHH in the inner rings, with detailed contexts in the outer rings). 5gmC sites in CC125 and CC5325 strains were determined by DEA-seq and TET-BS-seq. 5mC abundance was calculated by subtracting 5gmC from WGBS data, denoted as ‘BSΔDEA’ or ‘BSΔTET-BS’, respectively. (**C**) Genomic contexts of 5gmC distribution in DNA. (**D**) Modification levels of 5mC and 5gmC in distinct C-contexts. (**E**) Frequency distribution of 5mC and 5gmC in different C-contexts (*y*-axis shown on a log10 scale). (**F**) Average ratio and standard deviation of 5mC and 5gmC at CpG and non-CpG sites. All the ratios discussed above were calculated based on confidently identified 5mC and 5gmC sites, and the average ratios within these sites were determined.

In contrast to mammalian cells, where 5mC is predominantly found in CpG contexts [44], 5mC in *C. reinhardtii* is present at both CpG and non-CpG sites. Among the non-CpG contexts, CHH sites account for the largest proportion of total C-contexts, with over 46% of 5mC sites located in this context. The CHG context harbours about half as many 5mC sites as CHH contexts (Fig. 2A). However, when the 5mC sites were normalized to the total number of each C-context, CpG sites were found to be the most enriched in 5mC. Additionally, 5mC also exhibits a preferential distribution in CpA contexts (Supplementary Fig. S7B).

In comparison, DEA-seq and TET-BS-seq analysis revealed that 5gmC is less enriched at CpG sites and instead exhibited a preferential distribution in CHH contexts, suggesting that 5gmC plays a distinct role compared to 5mC (Fig. 2B and Supplementary Fig. S7B). Notably, 5gmC identified from TET-BS-seq appeared more frequently at CpG sites like 5mC. This might be due to insufficient 5mC to T conversion efficiency, further highlighting the advantage of DEA-seq in accurately profiling 5gmC (Fig. 2B). Furthermore, background noise signals detected in the *Dnmt1* knockout strain showed a 5gmC distribution pattern similar to that of 5mC, and didn't exhibit any preference for various genomic contexts according to DEA-seq, underscoring the confounding effect of residual C or 5mC in 5gmC detection and emphasizing the importance of using negative controls to eliminate false-positive signals (Supplementary Fig. S7B-E).

No significantly enriched motifs for 5gmC were detected in either sequencing approach (Fig. 2C and Supplementary Fig. S8A), albeit 5gmC seems to be more enriched at CAC or CCA contexts (Supplementary Fig. S7B). Moreover, the modification level of 5gmC appears higher at CHH contexts, while 5mC is more abundant at CpG sites similar to previously reported data (Fig. 2D and Supplementary Fig. S8B) [45, 46]. High 5mC ratio sites are spread across CpG sites, whereas more sites with high 5gmC ratio were found at CHH contexts (Fig. 2E, F and Supplementary Fig. S8C, D), emphasizing the unique role of 5gmC as an epigenetic mark independent of 5mC. Both CC125 and CC5325 strains of *C. reinhardtii* exhibited similar patterns of 5mC and 5gmC distribution (Fig. 2 and Supplementary Fig. S8), suggesting a conserved role of both DNA modifications across different strains.

### 5gmC is enriched in the intronic regions

**Figure 3.**
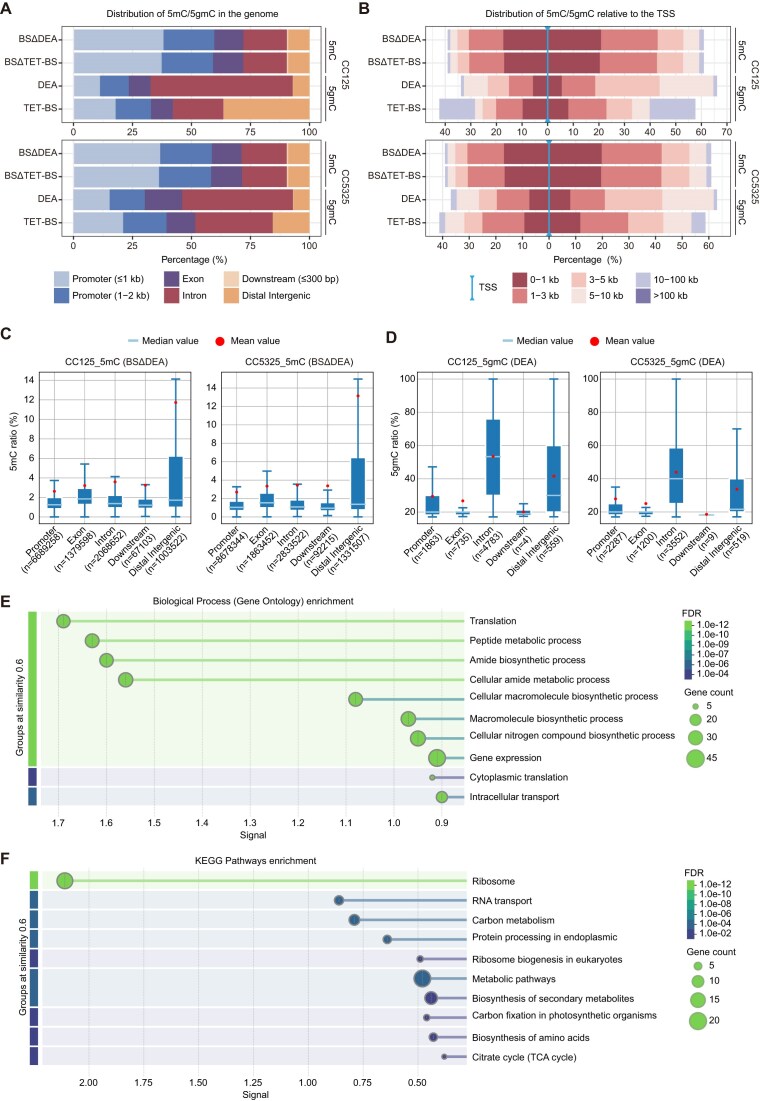
Distribution of 5gmC in functional regions across the genome. (**A**) Site-specific distribution of 5mC and 5gmC across different functional regions of the genome. (**B**) Percentage of 5mC or 5gmC at various distances upstream and downstream of the TSS. (**C, D**) Modification levels of 5mC (**C**) and 5gmC (**D**) in different functional regions. The ratios were calculated based on confidently identified 5mC and 5gmC sites, and the average ratios within these sites were determined. (**E, F**) GO (**E**) and KEGG (**F**) pathway analysis of genes with 5gmC located in promoter (≤2000 bp), exon, intron, or downstream (≤300 bp) regions.

In mammalian cells, CpG islands within the promoter regions of protein-coding genes are generally less methylated, whereas gene bodies maintain high methylation levels, highlighting the distinct roles of 5mC in different functional regions [6]. In *C. reinhardtii*, we found that approximately half of the 5mC sites are located in the promoter regions upstream of the transcription start site (TSS) (Fig. 3A), with over 70% of 5mC sites falling within 3 kb of the TSS (Fig. 3B). Furthermore, 5mC distribution across various C-contexts shows a similar pattern in these functional regions (Supplementary Fig. S9A, B).

According to DEA-seq, 5gmC sites are more preferentially located in the intronic regions, while exhibiting lower occupancy in promoters or around TSS regions (Fig. 3A, B). Notably, this enrichment of 5gmC within introns predominantly arises from CHH contexts (Supplementary Fig. S9A, B). TET-BS-seq partially supports these findings in both strains, further validating the observed distribution pattern. Additionally, while 5mC density is highest in the distal intergenic regions (Fig. 3C and Supplementary Fig. S9C), 5gmC shows its highest ratio in introns and distal intergenic regions (Fig. 3D and Supplementary Fig. S9D). TET-BS-seq also shows high 5gmC level in promoters (Supplementary Fig. S9D), likely due to the high occupancy of 5mC in promoters and insufficient conversion of 5mC to T. 5gmC was significantly enriched in genes related to metabolic and biosynthetic pathways (Fig. 3E, F), indicating a potential role of 5gmC in photosynthesis as previously reported [20].

### Distribution of 5gmC at transposable elements

Repetitive elements constitute more than 50% of the mammalian genome, with a large proportion of 5mC localized to transposable elements (TE), where 5mC is known to play a role in silencing transcriptional activity [6]. In contrast, 5mC is more enriched in gene bodies rather than in repeats in *C. reinhardtii* [45]. Similarly, our findings reveal that approximately 17% of 5mC is found within 1 kb regions upstream or downstream of TE (Supplementary Fig. S10A). Interestingly, 5mC is rarely located directly within TE regions. In comparison, a slightly larger proportion of 5gmC sites were detected within TE (Supplementary Fig. S10B). Although the number of confident 5mC sites within TE is relatively low, the ratio of 5mC is significantly higher within TE than in other genomic regions (Supplementary Fig. S10C). In contrast, 5gmC levels appear similar between TE and other transcribed genes according to DEA-seq analyses (Supplementary Fig. S10D). These findings suggest that while 5mC plays a conserved repressive role in TE across species, the presence of 5gmC may counteract this repression, potentially alleviating the silencing effects within these regions.

### 5gmC levels are inversely correlated with 5mC levels in the genome

**Figure 4.**
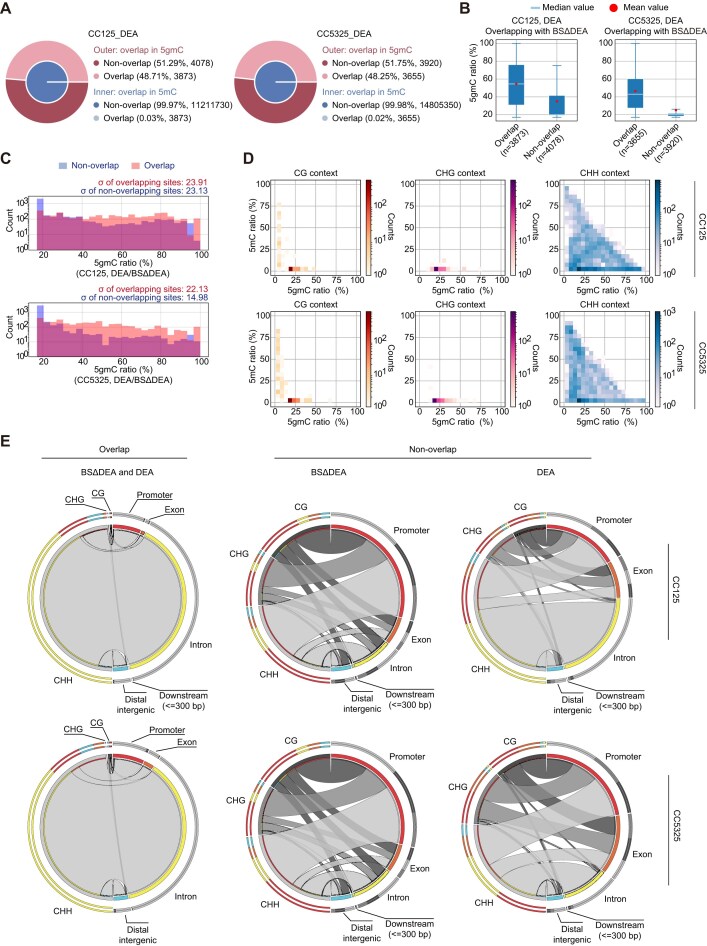
Correlation between 5gmC and 5mC. (**A**) Overlap analysis of confident 5gmC and 5mC sites identified by DEA-seq and WGBS. (**B**) Comparison of 5gmC modification levels at overlapping and non-overlapping sites between 5mC and 5gmC. The ratios were calculated based on confidently identified 5gmC sites, and the average ratios within these sites were determined. (**C**) Distribution of 5mC and 5gmC counts (*y*-axis shown on a log10 scale) at overlapping and non-overlapping sites. σ denotes the standard deviation. (**D**) 5mC and 5gmC levels at confident 5gmC sites across different genomic contexts. (**E**) Site-specific distribution of 5gmC and 5mC in CG, CHG, and CHH contexts within various functional genomic regions. Both DNA modifications at the overlapping and non-overlapping sites are shown separately.

5gmC originates from vitamin C-modified 5mC, but the fate of 5gmC remains unclear. To investigate this, we compared the distribution of confidently identified 5mC and 5gmC sites, and found that more than half of 5gmC sites does not overlap with 5mC, indicating mutual exclusivity between these two DNA modifications in the genome (Fig. 4A and Supplementary Fig. S11A). Interestingly, 5gmC is more abundant at the subset of overlapping sites (Fig. 4B and Supplementary Fig. S11B), and a larger proportion of 5gmC at these overlapping sites shows a higher modification level (Fig. 4C and Supplementary Fig. S11C). These suggest that 5gmC undergoes rapid turnover, facilitating efficient DNA demethylation at the non-overlapping sites. On the contrary, at the overlapping sites, the demethylation process appears to be ongoing, with 5gmC remaining at high level in combination with 5mC.

In terms of 5gmC distribution across different C-contexts, a negative correlation between 5gmC and 5mC is evident, with the 5mC ratio being lowest at CHG sites that contain 5gmC (Fig. 4D and Supplementary Fig. S11D). The overlapping sites are predominantly located at CHH contexts according to DEA-seq and TET-BS-seq (Fig. 4E and Supplementary Fig. S11E). Notably, overlapping CHH sites are mainly found in introns. In contrast, the non-overlapping 5gmC sites to some extent follow a similar distribution pattern to 5mC, with the majority of 5gmC located at promoter or intronic regions (Fig. 4E and Supplementary Fig. S11E).

### 5gmC is correlated with active gene transcription

**Figure 5.**
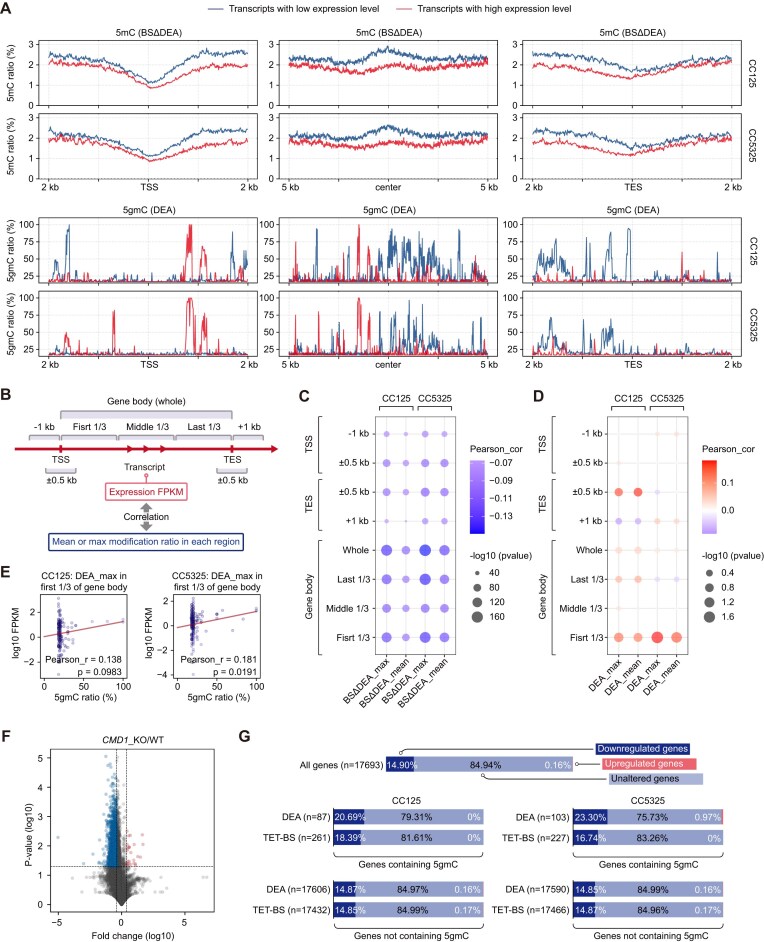
Correlation between 5gmC and active gene transcription. (**A**) Distribution of 5mC and 5gmC around the TSS, gene body, and transcription end site (TES) of differentially transcribed genes. Genes were categorized as highly transcribed (top 25% by FPKM) or lowly transcribed (bottom 25% by FPKM), denoted by red or blue lines, respectively. (**B**) Schematic representation of the annotated regions for each transcript, which is divided into several parts. (**C, D**) Correlation between gene expression and 5mC (**C**) or 5gmC (**D**) levels in different regions as indicated in panel B. Modification levels were characterized using both the maximum (max) and mean values. (**E**) Dot plots showing the positive correlation between transcript levels and 5gmC in the first third of the gene body regions in CC125 and CC5325 cells. (**F**) Volcano plot showing the distribution of genes that are significantly downregulated or upregulated in the *CMD1* mutant strain. (**G**) Proportion of genes with or without 5gmC that are upregulated or downregulated upon *CMD1* depletion.

DNA modifications play crucial roles in regulating gene expression, with different modifications often exerting opposing effects. Among these, 5mC is primarily known for its role in repressing gene expression in mammals and many other organisms [2], while 6mA is suggested to be positively correlated with gene expression [10, 47]. In this study, we separated protein-coding genes into different categories based on their expression levels and observed that actively transcribed genes contain lower levels of 5mC, particularly in the gene body regions (Fig. 5A and Supplementary Fig. S12A). While the levels of 5gmC did not show a significant difference between highly expressed and lowly expressed genes globally, 5gmC marks actively transcribed genes particularly in the upstream regions of gene bodies, as revealed by DEA-seq data (Fig. 5A).

To further explore the relationship between DNA modification levels and gene expression, we divided the gene structure into several functional regions and assessed the correlation of DNA modifications with gene expression levels (Fig. 5B). As expected, the presence of 5mC in gene bodies and other regulatory regions was negatively correlated with gene expression (Fig. 5C and Supplementary Fig. S12B), a pattern that contrasts with the positive role of gene body 5mC in gene expression observed in mammals [7]. In contrast, 5gmC showed a positive correlation with gene expression, particularly within upstream regions of gene bodies, a pattern consistently observed in both DEA-seq and TET-BS-seq datasets (Fig. 5D, E and Supplementary Fig. S12C, D). Notably, two representative 5gmC loci within gene bodies are highlighted (Supplementary Fig. S12E), illustrating this potential correlation between 5gmC presence and gene transcription.

Additionally, analysis of *CMD1* knockout samples from our previous study revealed that numerous genes were downregulated following *CMD1* depletion (Fig. 5F) [20]. Moreover, among the genes containing 5gmC, more than 20% were downregulated upon *CMD1* depletion, while almost none were upregulated (Fig. 5G). By contrast, only ∼14% of genes lacking 5gmC were downregulated, further supporting a positive regulatory role of 5gmC in gene expression. Notably, since only ∼0.5% of total genes contain 5gmC, its effect on promoting transcription is likely restricted to this limited subset of the genome.

### The crosstalk between 5gmC and other epigenetic modifications

DNA modifications are known to interact with other epigenetic marks, such as histone modifications and RNA methylation, to regulate gene expression and chromatin structure in a variety of organisms, including mammals [5, 48]. In *C. reinhardtii*, 6mA marks distinct regions from 5mC, indicating that these DNA modifications may play complementary roles in the regulation of gene expression [49]. In this study, we explored the potential crosstalk between 5gmC and other chromatin modifications, with a focus on histone lysine methylation, using published data on histone modifications in *C. reinhardtii* [28]. Histone H3 modifications, particularly H3K4me3 (trimethylation of histone H3 on Lys 4) and H3K9me3 (trimethylation of histone H3 on Lys 9), are well-studied in mammals as markers of transcriptionally active and silent chromatin, respectively [5]. However, H3K4me3 was shown to be maintained independently of gene expression, and the roles of them in *C. reinhardtii* remain under investigation [50]. On the other hand, H3K9me1 (monomethylation of histone H3 on Lys 9) has been associated with transcriptional repression in *C. reinhardtii* [28, 51].

**Figure 6.**
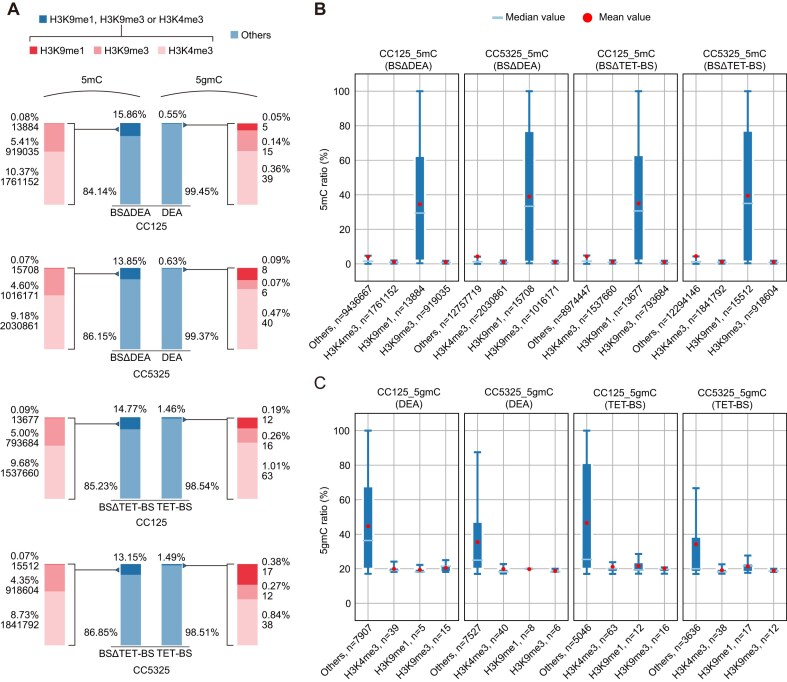
Crosstalks between 5gmC, 5mC, and histone methylation. (**A**) Percentage and counts of 5mC and 5gmC sites marked by specific histone modifications. For the overlapping sites between DNA modifications and histone modifications, the proportion and counts of 5mC and 5gmC in genomic regions associated with H3K9me1, H3K9me3, and H3K4me3 were also calculated. (**B**and**C**) Modification levels of 5mC (**B**) or 5gmC (**C**) across regions marked by different histone modifications.

Our analysis showed that approximately 15% of 5mC sites overlap with regions marked by histone H3 methylation examined in this study, with a notable preference for H3K4me3 sites (Fig. 6A). Interestingly, while 5mC is rarely found in H3K9me1-marked regions, DNA associated with H3K9me1 exhibited a remarkably high level of DNA methylation, suggesting a synergistic repressive role between 5mC and H3K9me1 in *C. reinhardtii* (Fig. 6B). In contrast, 5gmC was found to be much less prevalent within these regions marked by histone methylation (Fig. 6A) and did not show any significant enrichment for H3K9me1 or other histone methylation sites (Fig. 6C), which contrasts with the repressive roles of 5mC in the genome. This pattern is consistent with the enrichment of 5gmC in intronic regions, which may be largely devoid of these histone modifications.

## Discussion

In this study, we introduced DEA-seq, a novel and efficient method for distinguishing 5gmC from C and 5mC across the genome using a one-step DNA deaminase treatment before sequencing. Using DEA-seq, we successfully mapped the distribution of 5gmC across the genome of *C. reinhardtii* and characterized 5mC sites by subtracting 5gmC from WGBS data. This approach allows for the comprehensive profiling of both DNA modifications.

Our findings revealed that 5gmC is substantially enriched in CHH contexts, in contrast to the strong preference of 5mC for CpG sites. A greater proportion of 5gmC sites were found within intronic regions, while fewer 5gmC were identified around TSS regions. The highest ratio of 5gmC sites occurred in introns and distal intergenic regions, while 5mC was predominantly enriched in intergenic regions. Strikingly, more than half of the 5gmC sites were mutually exclusive from 5mC, suggesting distinct functional roles and inverse relationship between these two DNA modifications. Consistent with its enrichment in introns, 5gmC is positively correlated with gene expression. This is in stark contrast to 5mC, which generally exerts suppressive effects, regardless of its locations in gene body or regulatory regions. 5mC appears to synergize with H3K9me1 to exert its repressive effects. However, the conversion of 5mC to 5gmC alleviates the suppressive effects on gene expression, as 5gmC didn't show cooperative effects with these histone methylation marks. All of the discoveries identified are consistent in two *C. reinhardtii* strains (CC125 and CC5325), although they share different genetic backgrounds.

In DEA-seq, only one commercial deaminase mix is used to treat DNA before library construction and next-generation sequencing. Compared to the previously proposed TET-BS-seq, the DEA treatment is milder, as it does not fragment DNA into short pieces, allowing for the use of smaller amounts of DNA. This makes it particularly useful for trace samples or single-cell sequencing. Moreover, while TET-mediated oxidation of 5mC into 5hmC is most efficient, further oxidation into 5fC and 5caC is largely inhibited, requiring additional enzymes [52]. In contrast, the deaminase activity of DEA is potent enough to convert both C and 5mC into U or T, and this activity is completely blocked by the glyceryl moiety of 5gmC. Thus, the DEA-seq could be used not only to distinguish 5gmC but also to detect other potential hypermodified cytosines in various organisms. Although deaminase-based methods have been developed to profile 5mC and 5hmC [53, 54], they cannot effectively distinguish these modifications due to their co-occurrence in the mammalian genome. This highlights the importance of both the deaminase enzyme employed in the sequencing and the diversity of DNA modifications in the species that to be studied. Our DEA-seq method, applied in *C. reinhardtii*, leverages the unique presence of only 5mC and 5gmC in the genome, enabling direct and specific detection of 5gmC without interference from other hypermodified cytosine bases.

One of the most interesting findings in this study is the exclusivity observed between many 5gmC sites and 5mC. Although 5gmC sites are much fewer than 5mC sites, this suggests that 5mC at specific loci is efficiently and completely converted into 5gmC by CMD1. A previous study has reported a modest CpG preference for CMD1 activity [21], however, in our work, a greater proportion of 5gmC was observed to be located at CHH sites. This implies that 5gmC at CpG sites may undergo rapid turnover, thereby promoting active DNA demethylation at these sites and maintaining a low steady-state level of 5gmC in the genome. This interpretation is also supported by the observed increase in 5mC at CpG loci upon CMD1 depletion, reinforcing the role of 5gmC in facilitating DNA demethylation specifically at CpG sites [20]. However, the enzymes of pathways responsible for removing 5gmC remain unidentified. A detailed study of DNA glycosylases in *C. reinhardtii* may help to further elucidate the active DNA demethylation pathway, akin to what has been observed in mammals [55, 56]. Additionally, the enzymatic activity of CMD1 *in vivo* may be influenced by the cellular microenvironment, including the presence of interacting proteins and chromatin context, which could modulate CMD1 targeting and activity.

The mutual exclusion between 5gmC and 5mC also suggests that they likely have distinct roles in the genome, with 5gmC occupying specific loci where 5mC is absent. Indeed, 5gmC is enriched in intronic regions. Although 5gmC marks only a small proportion of protein-coding genes, it is positively correlated with gene transcription. Since 5mC is shown to suppress gene expression regardless of its location in gene bodies or promoters, active DNA demethylation mediated by 5gmC may result in the reactivation of transcription. Furthermore, the specific enrichment of 5gmC in gene bodies, suggests that 5gmC might also serve as a stable epigenetic mark to regulate gene expression independent of 5mC, reminiscent of the roles of 5hmC in transcription [57, 58]. Given this, the role of 5gmC warrants further investigation. For instance, identifying the specific reader proteins of 5gmC could facilitate understanding its regulatory roles in transcription.

Although 5gmC is less stable compared to 5mC *in vivo* [20], it marks certain genes in intronic regions. One remaining question is how 5gmC at these loci is maintained. This might be accomplished by the protection of specific 5gmC readers, which could prevent the binding of eraser enzymes. Alternatively, the specific recruitment of CMD1 to the introns could explain the persistence of 5gmC at these sites. To further clarify these mechanisms, the interactome of CMD1 proteins should be analysed.

The interplay between various epigenetic marks remains largely unexplored. The strong interaction between 5mC and H3K9me1 underscores their synergistic roles in repressing gene expression. Our recent study has demonstrated that depletion of Dnmt1, a key DNA methyltransferase in *C. reinhardtii*, leads to the loss of 5mC, which in turn results in a substantial reduction in H3K9me1 levels [28]. This observation suggests that H3K9me1 is generated and maintained by DNA methylation through mechanisms that remain to be fully elucidated. Interestingly, we found that 5gmC showed no preferential association with these histone methylation marks, which aligns with its enrichment in intronic regions that may lack such regulatory modifications. Thus, the conversion of 5mC into 5gmC potentially leads to the reactivation of previously silenced genes. Nevertheless, further investigation is warranted to clarify the relationship between 5gmC and other histone modifications and to better elucidate the complex interplay between DNA and histone modifications in gene regulation in *C. reinhardtii*. Additionally, 6mA has been shown to be enriched near TSS, and correlated with actively transcribed genes in *C. reinhardtii* [49], suggesting a potentially contrasting regulatory role between 5mC and 6mA that merits deeper exploration.

The distribution of 5gmC in other organisms remains largely unexplored. Although CMD1 has been identified as a homologue of TET proteins, which are found across many kingdoms, there are currently no reports documenting the presence of 5gmC in other species. DEA-seq offers a promising approach to accurately map 5gmC in other organisms, providing a powerful complement to mass spectrometry techniques. However, one limitation is that the deaminase activity of DEA may be blocked by other hypermodified cytosines, making it difficult to distinguish 5gmC from these other modifications if they co-exist in the same genome. Since DEA only deaminates cytosine derivatives, further exploration is needed to identify additional enzymes capable of detecting other modified bases specifically, such as 6mA [59].

In conclusion, our work not only presents DEA-seq as an innovative and straightforward method for profiling 5gmC, but also provides a comprehensive genome-wide landscape of 5gmC as well as 5mC in *C. reinhardtii*. This resource will be valuable for future studies on the roles of DNA modifications in green algae and potentially other organisms.

## Supplementary Material

gkaf955_Supplemental_File

## Data Availability

All data of this study are available in the main text or the supplementary materials. Additional materials and detailed protocols are available from the corresponding author upon reasonable request. The WGBS, TET-BS-seq, and DEA-seq data was deposited at GEO under accession number: GSE301796. The RNA-seq data was deposited at GEO under accession number: GSE301797. The DEA-seq, TET-BS-seq, WGBS and RNA-seq data have been deposited in the UCSC Genome Browser, which can be accessed at the website https://genome.ucsc.edu/s/Wang%20Fanchen/ 5gmC_WGBS%26RNA%2Dseq.
