# Supplementary material for: Base-resolution profiling of 5-glyceryl-methylcytosine in Chlamydomonas reinhardtii via deaminase-assisted sequencing
Source: Nucleic Acids Res. 2025 Sep 24;53(18):gkaf955. doi: 10.1093/nar/gkaf955 (PMC12458080; doi:10.1093/nar/gkaf955)
Supplement: gkaf955_Supplemental_File [file gkaf955_supplemental_file.pdf]

MANUSCRIPT TITLE

Base-resolution profiling of 5-glyceryl-methylcytosine in *Chlamydomonas reinhardtii* via deaminase-assisted sequencing

AUTHORS

Fan-Chen Wang[1,†], Bao-Dan He[1,†], Zi-Xin Wang[1], Xuan Deng[2], Hui Chen[3], Wei-Ying Meng[1], Yu-Tao Fu[1], Wan-Yue Zou[1], Tong Ge[3], Yawen Li[3,4], Shu-Xia Sun[1], Ke-Yao Zhao[1], Hao-Ming Jiang[1], Zhi-Yan Sun[1], Guo-Liang Xu[3], Kai-Yao Huang[2] and Jian-Huang Xue[1,*]

[1] Key Laboratory of Spine and Spinal Cord Injury Repair and Regeneration of Ministry of Education, Tongji Hospital affiliated to Tongji University, Frontier Science Center for Stem Cell Research, School of Life Sciences and Technology, Tongji University, Shanghai, 200092, China.
[2] Key Laboratory of Algal Biology, Institute of Hydrobiology, Chinese Academy of Sciences, Wuhan 430072, China.
[3] Key Laboratory of Epigenetic Regulation and Intervention, Chinese Academy of Sciences Center for Excellence in Molecular Cell Science, Shanghai Institute of Biochemistry and Cell Biology, Chinese Academy of Sciences, University of Chinese Academy of Sciences, Shanghai 200031, China.
[4] School of Life Science and Technology, ShanghaiTech University, Shanghai, 201210, China.

† The first two authors should be regarded as Joint First Authors.

* To whom correspondence should be addressed. Email: xuejianhuang@tongji.edu.cn.

The supplementary material includes 12 figures.

Supplementary Figures and Figure legends

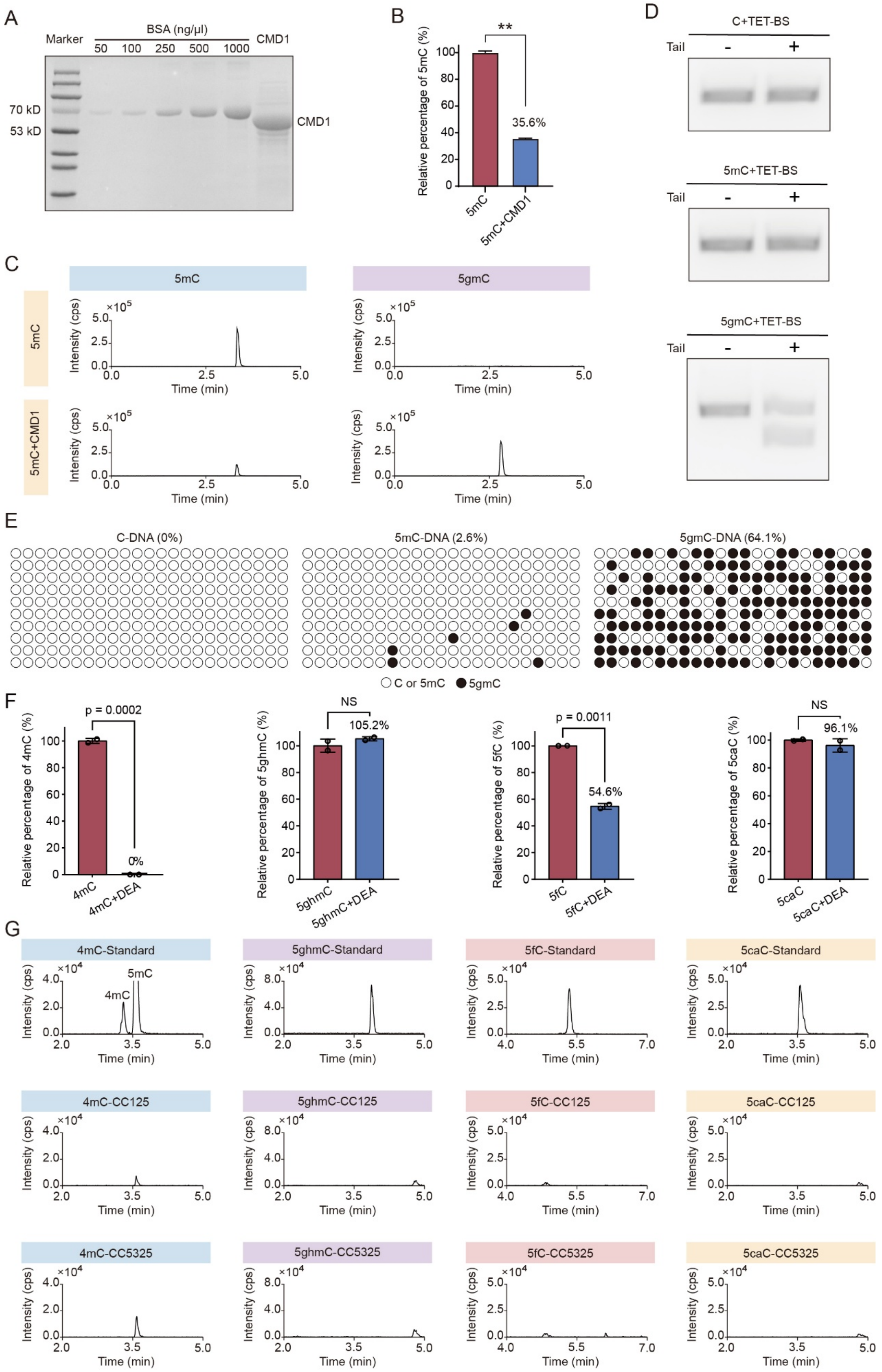

Supplementary Figure S1. Validation of TET-BS-seq. (**A**) Coomassie blue staining of purified recombinant CMD1 proteins from E. coli. (**B**) Representative images showing the MS analysis of 5gmC generated by CMD1-treated methylated lambda DNA. (**C**) Quantification of 5mC levels after CMD1 treatment. Data are mean ±SD from two independent biological replicates. (**D**) Restriction enzyme digestion analysis of differently modified DNA after TET and bisulfite treatment, followed by PCR amplification. (**E**) Sanger sequencing analysis of lambda DNA containing different modified cytosines after TET and bisulfite treatment. Each circle represents a CpG site in the lambda DNA sequence. (**F**) MS quantification of different DNA modifications following treatment with the deaminase mix used in this study. The DNA modifications analyzed include 4mC, 5ghmC, 5fC, and 5caC. Data are presented as mean ±SD from two independent biological replicates. (**G**) MS analysis of various DNA modifications in the genome of *C. reinhardtii*. Representative MS results for 4mC, 5ghmC, 5fC, and 5caC from two biological replicates are shown.

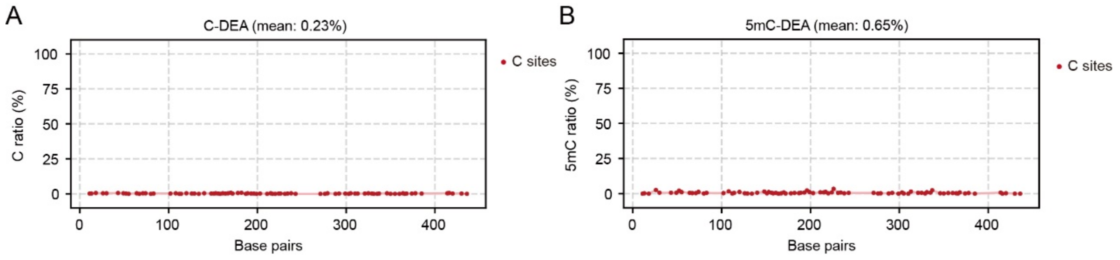

C

Contexts	Site number	Total C	Deaminated C	Conversion rate (%)
CpG	9	4518	4506	99.73
CHG	26	13043	13018	99.81
CHH	47	24107	24050	99.76

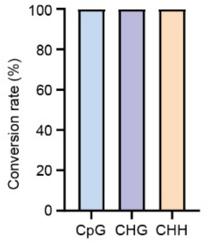

D

Contexts	Site number	Total 5mC	Deaminated 5mC	Conversion rate (%)
CpG	9	6331	6292	99.38
CHG	26	17595	17433	99.08
CHH	47	31114	30958	99.50

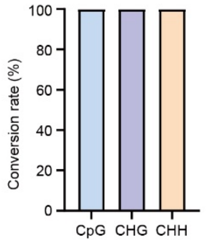

Supplementary Figure S2. The conversion rates of C and 5mC in various genomic context upon treated with DEA enzyme mix. (**A**) The remaining cytosines after DEA treatment. Each site represents a cytosine, in CpG or non-CpG sites, of the spike-in DNA fragment. (**B**) The remaining 5mC after DEA treatment. Each site represents a 5mC, in CpG or non-CpG sites, of the spike-in DNA fragment. (**C, D**) The summarized conversion rates for unmodified cytosines (**C**) or 5mC (**D**) in different genomic contexts as shown in panel A and B. Representative results are shown from two independent replicates.

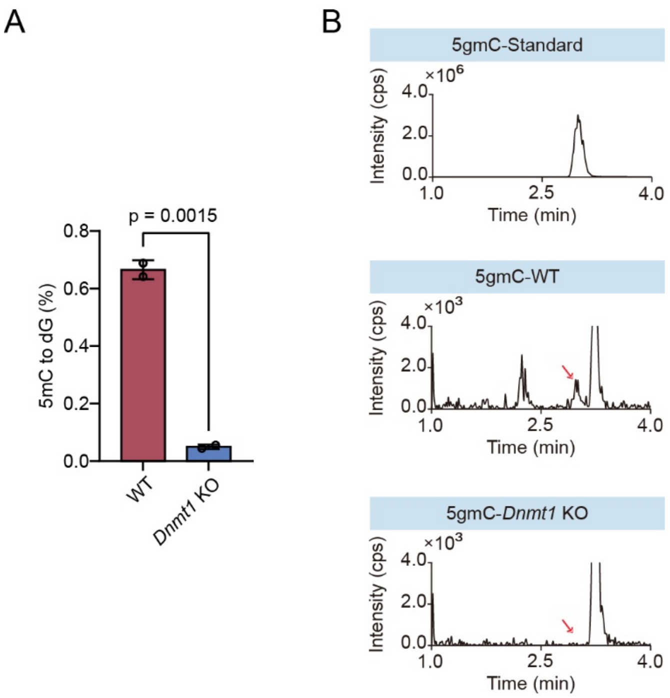

Supplementary Figure S3. 5mC and 5gmC levels in the *Dnmt1* KO strain of *C. reinhardtii*. (**A**) MS quantification of 5mC in the genome. (**B**) Representative image showing the 5gmC peaks in the genome of *C. reinhardtii*.

A

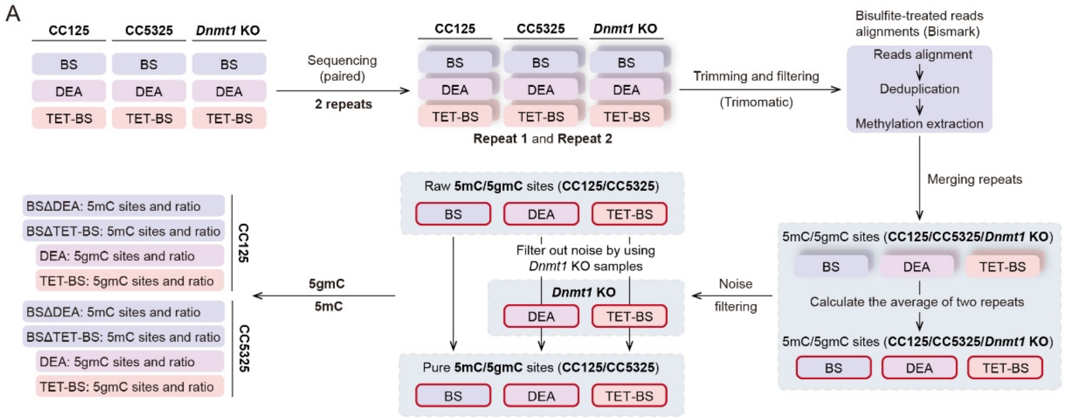

B

Sample	Avg dups (%)	Avg seqs (M)	Avg depth (×)	Coverage (%, depth>0×)	Coverage (%, depth>10×)
CC125_BS1	21.50	47.29	98.72	91.97	88.39
CC125_BS2	22.96	46.05	98.11	91.87	88.28
CC125_DEA1	26.89	50.48	98.09	91.99	88.26
CC125_DEA2	28.15	56.46	106.25	92.02	88.46
CC125_TET-BS1	25.33	45.34	93.29	91.62	86.48
CC125_TET-BS2	28.65	66.14	129.70	91.93	87.81
CC5325_BS1	20.51	47.23	96.10	91.21	87.23
CC5325_BS2	24.33	71.92	145.39	91.55	88.00
CC5325_DEA1	24.79	49.91	94.83	91.18	86.82
CC5325_DEA2	26.65	63.74	116.15	91.46	87.80
CC5325_TET-BS1	25.36	48.86	95.90	90.86	85.04
CC5325_TET-BS2	26.92	58.09	111.98	91.08	86.08
Dnmt1 KO_BS1	20.54	56.12	121.85	91.33	87.61
Dnmt1 KO_BS2	20.24	51.43	110.56	91.24	87.36
Dnmt1 KO_DEA1	22.13	47.04	96.31	90.90	86.71
Dnmt1 KO_DEA2	20.93	48.31	98.59	91.17	87.31
Dnmt1 KO_TET-BS1	22.81	63.14	126.39	91.50	87.90
Dnmt1 KO_TET-BS2	23.79	65.94	130.15	91.44	87.88

C D

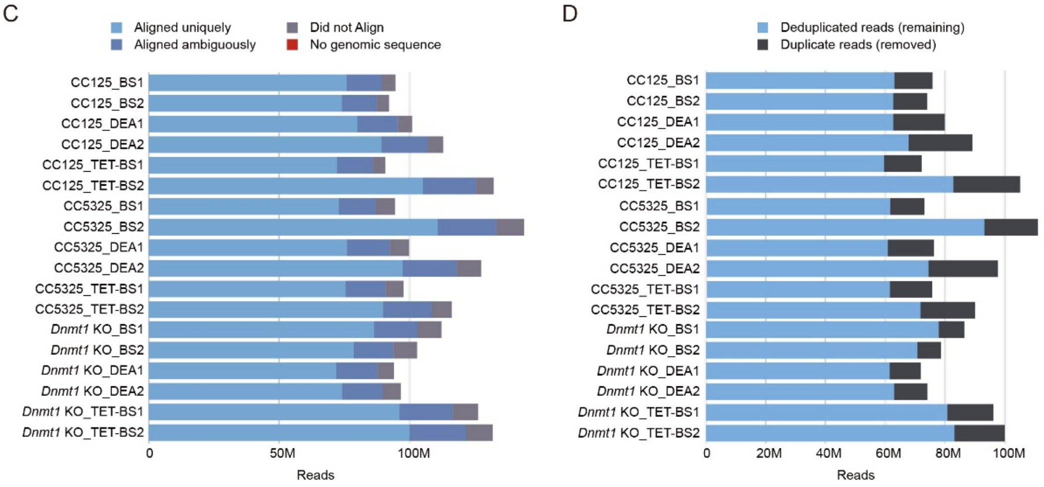

Supplementary Figure S4. Next-generation sequencing data analysis and quality control. (**A**) Flowchart of 5mC and 5gmC sequencing and data analysis pipelines. (**B**) Summary of filtered sequencing reads for WGBS, DEA-seq and TET-BS-seq, each including two biological replicates. (**C**) Reads alignment results obtained using Bismark software. (**D**) Reads deduplication results using Bismark software.

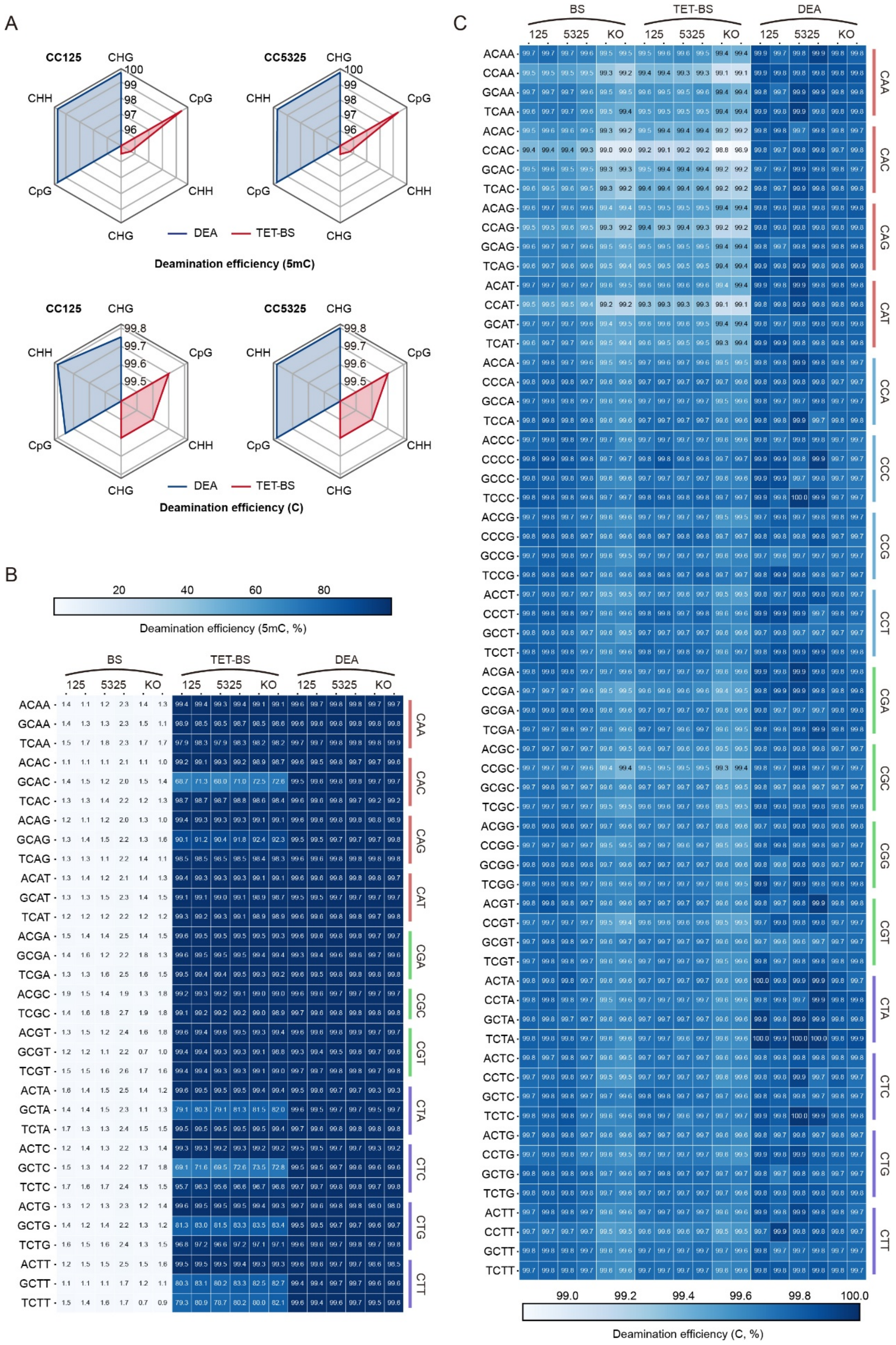

Supplementary Figure S5. DEA-seq exhibits high conversion efficiency of cytosines and 5mC across various genomic contexts without bias, comparing to TET-BS-seq. (**A**) Overall C-to-T conversion rates of 5mC in different sequence contexts

(CpG, CHG, CHH) of the 5mC spike-in DNA, as measured by DEA-seq and TET-BS-seq. (**B**) Conversion rates of 5mC in various genomic contexts of the 5mC spike-in DNA determined by WGBS, TET-BS-seq, and DEA-seq. (**C**) Conversion rates of unmodified cytosines in different genomic contexts of spike-in lambda DNA determined by WGBS, TET-BS-seq, and DEA-seq.

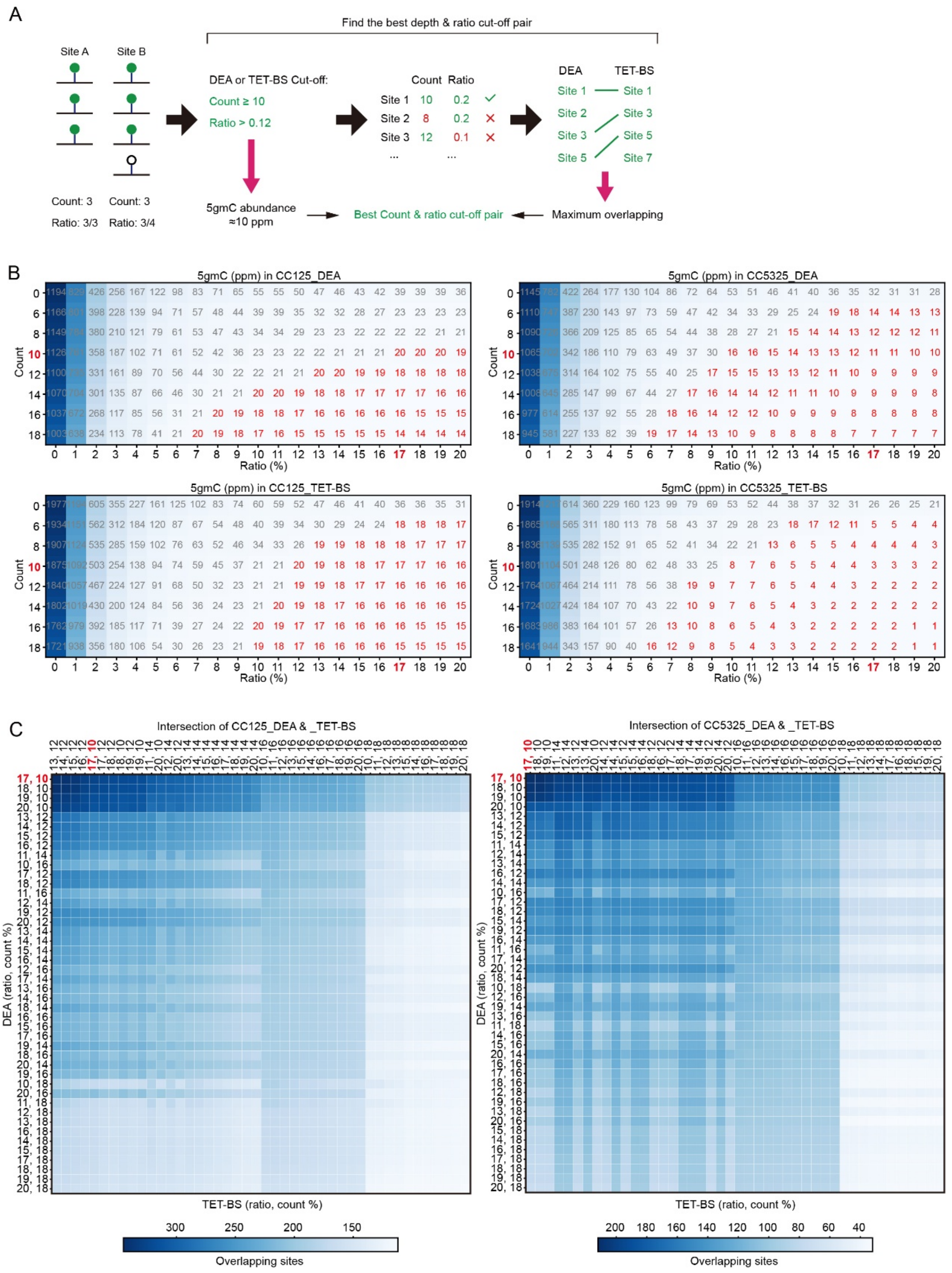

Supplementary Figure S6. Determination of the appropriate cut-off for confident 5gmC site acquisition. (**A**) Schematic representation of the process to determine the optimal cut-off in 5gmC sequencing data analysis. (**B**) 5gmC abundance calculation using different sequencing depths and ratios as cut-offs in DEA-seq and TET-BS-seq. (**C**) Overlapping 5gmC sites identified from DEA-seq and TET-BS-seq using various sequencing depths and ratios as cut-offs.

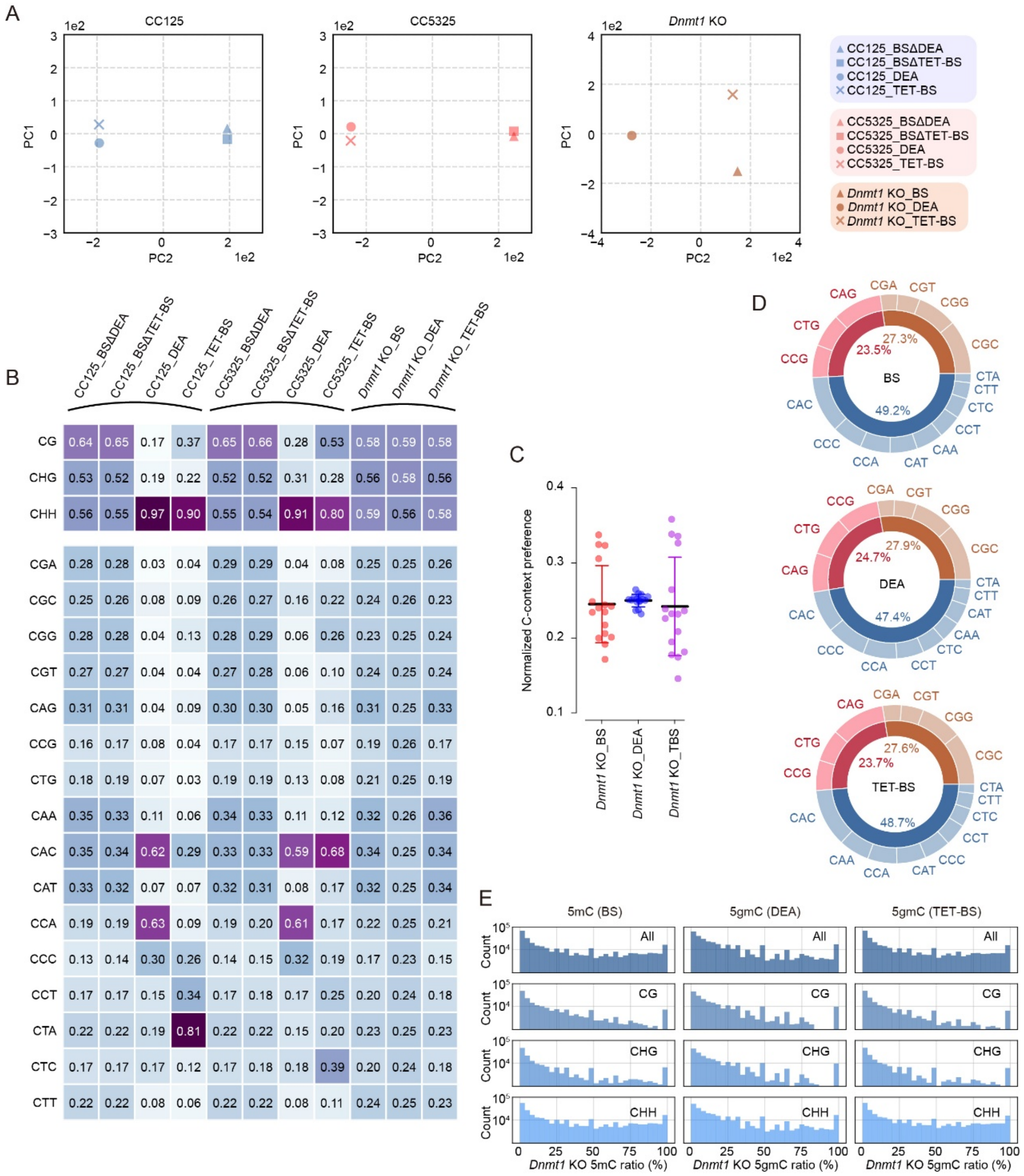

Supplementary Figure S7. 5gmC exhibits strong preference in CHH contexts in WT cells but not in *Dnmt1* KO strains. (**A**) Principal component analysis (PCA) of 5mC and 5gmC sequencing results in WT or *Dnmt1* KO strains. (**B**) Relative site-specific distribution propensity of 5mC and 5gmC in various C-contexts. The distribution propensity was normalized by the abundance of different genomic contexts, allowing for a comparative analysis of the site-specific distribution patterns. (**C**) Normalized context preference of different sequencing methods as detected in *Dnmt1* KO stains. (**D**) Distribution contexts of 5mC or 5gmC in genomic DNA detect by different sequencing methods in *Dnmt1* KO strains. (**E**) Distribution of 5mC and 5gmC counts (y-axis shown on a log10 scale) across different C-contexts in *Dnmt1* KO strains. Notably, 5gmC signals detected in the knockout strains were treated as background noise and subtracted from wild-type data to define authentic 5gmC sites.

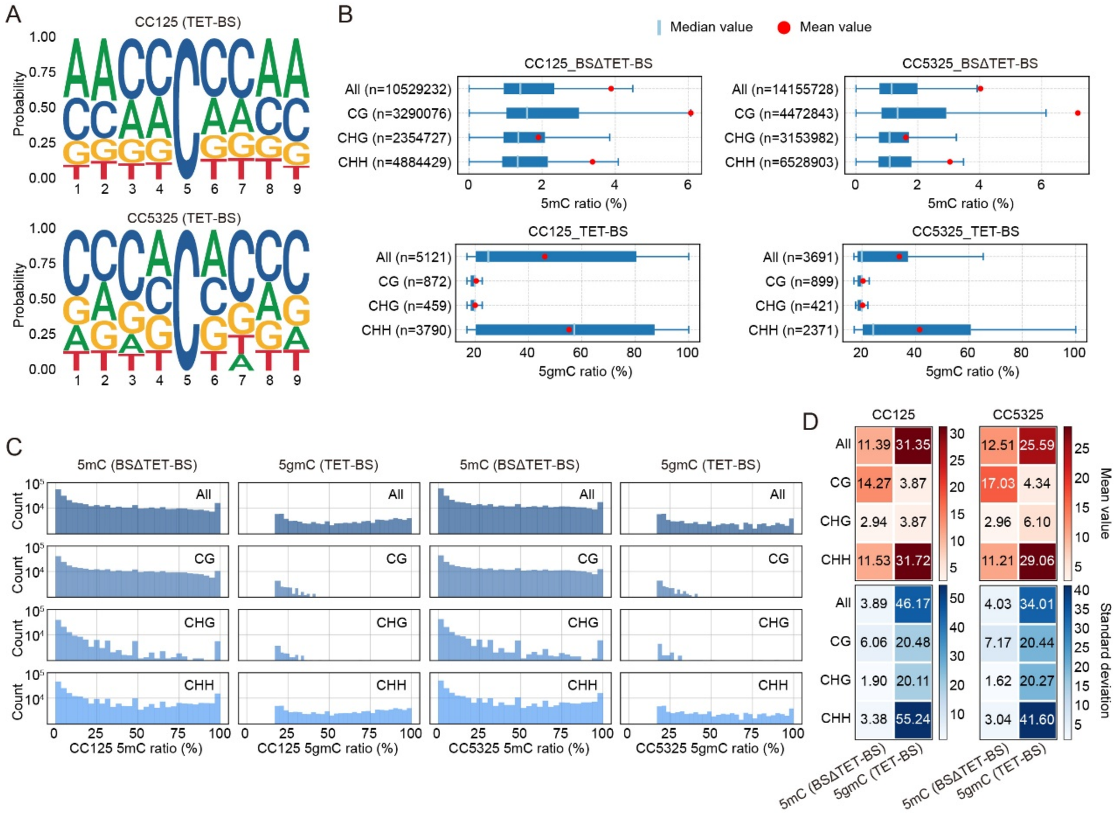

Supplementary Figure S8. Distribution of 5gmC in different C-contexts by TET-BS-seq. (**A**) Distribution contexts of 5gmC in genomic DNA. (**B**) Levels of 5mC and 5gmC in different C-contexts. Red dots represent mean values, while white lines indicate median values. (**C**) Distribution of 5mC and 5gmC counts (y-axis shown on a log10 scale) across different C-contexts. (**D**) Average ratio of 5mC and 5gmC, along with standard deviation values, in various C-contexts. All the ratios discussed above were calculated based on confidently identified 5mC and 5gmC sites, and the average ratios within these sites were determined.

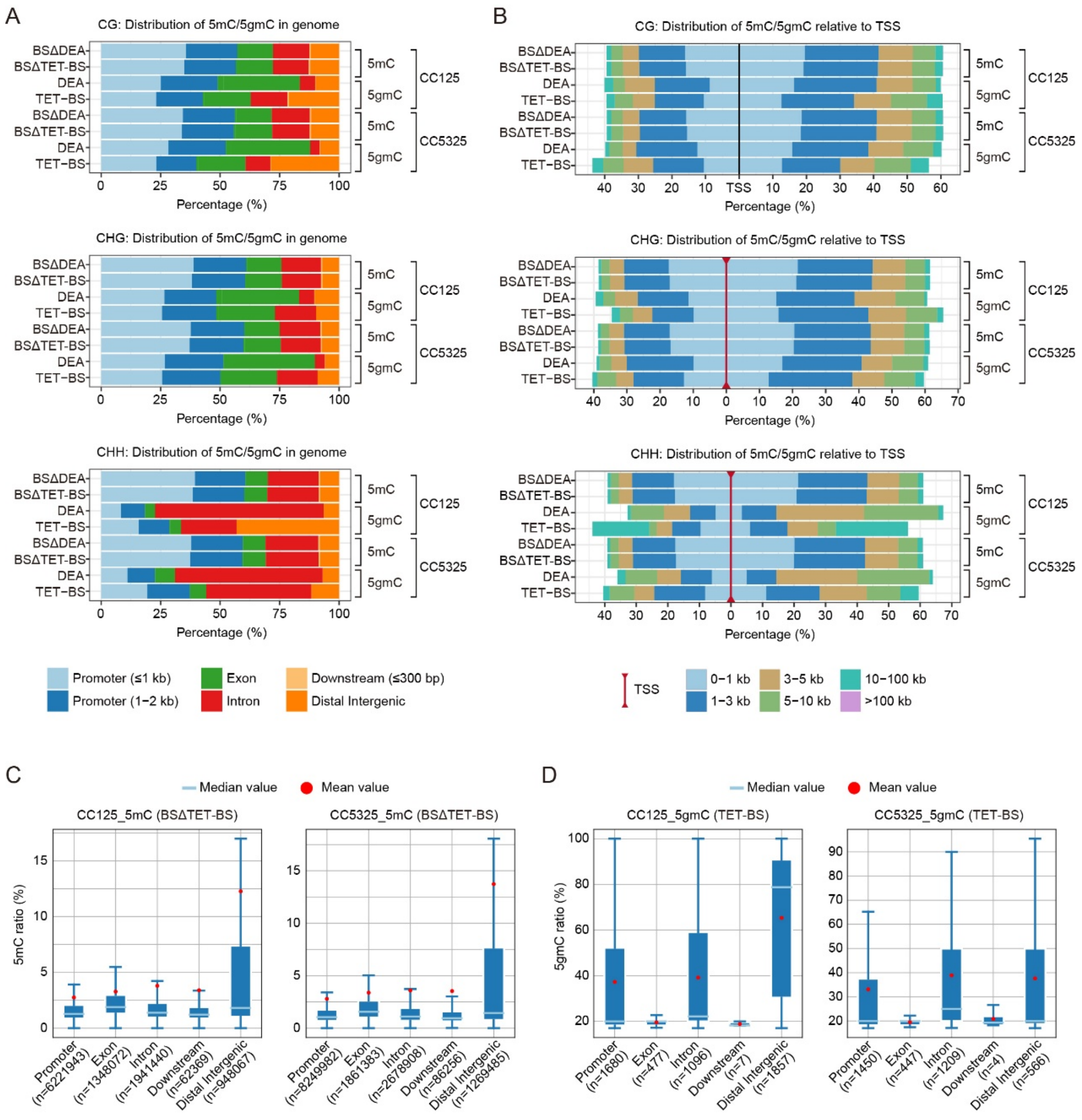

Supplementary Figure S9. Site-specific distribution of 5gmC in various C-contexts within different functional regions. (**A**) Site-specific distribution of 5mC and 5gmC in CG/CHG/CHH contexts within functional regions. (**B**) Percentage of 5mC or 5gmC in different C-contexts at varying distances upstream and downstream of the TSS. (**C, D**) Levels of 5mC (**C**) and 5gmC (**D**) across functional regions of the genome determined by TET-BS-seq. Red dots represent mean values, while white lines indicate median values. The ratios were calculated based on confidently identified 5mC and 5gmC sites, and the average ratios within these sites were determined.

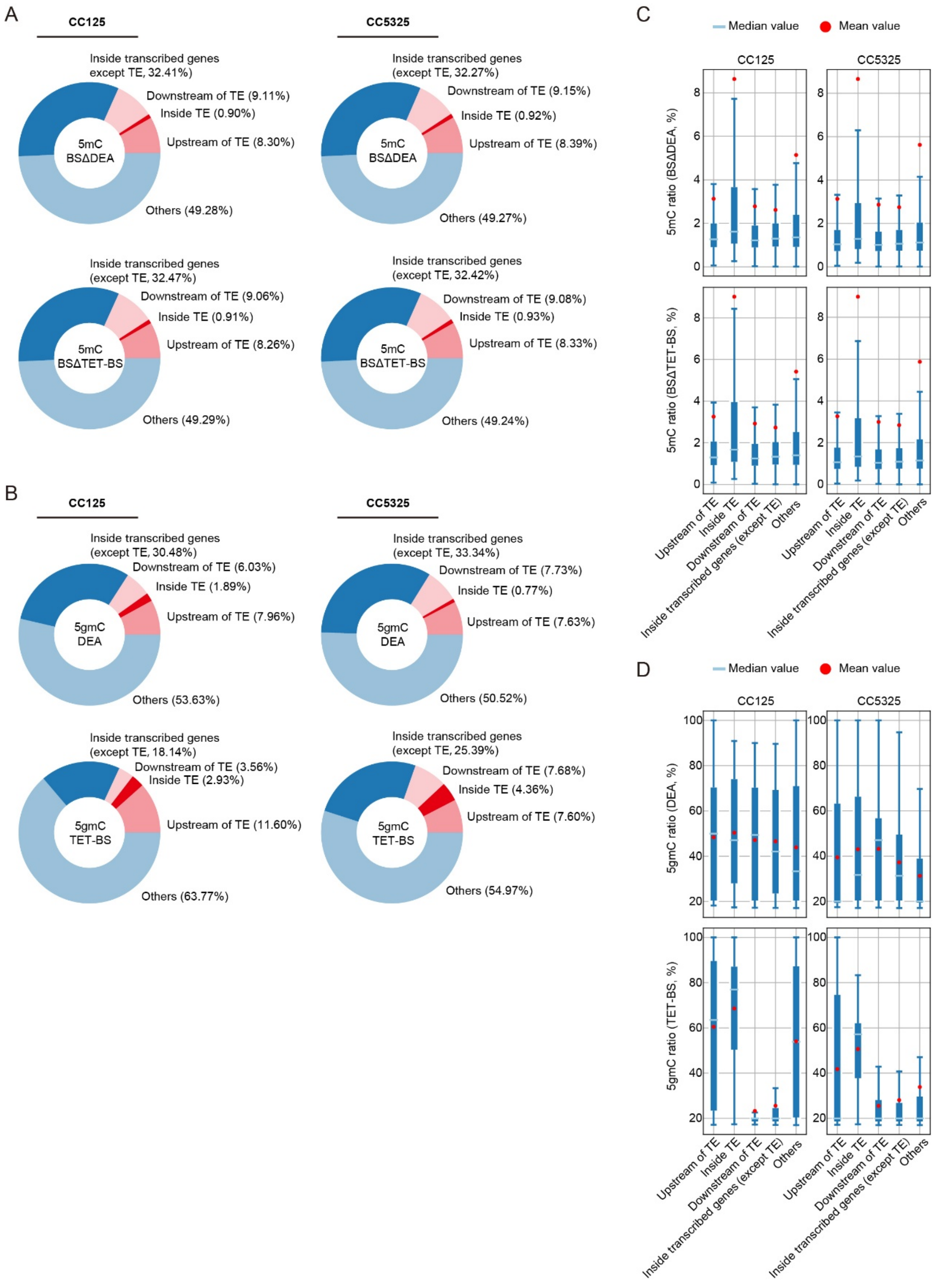

Supplementary Figure S10. Distribution of 5gmC in Transposable Elements. (**A**) Distribution of 5mC in TE regions and other transcribed genes. (**B**) Distribution of 5gmC in TE regions and other transcribed genes. (**C**) Modification levels of 5mC at various regions surrounding TE sites. Red dots represent mean values, while white lines indicate the median values. (**D**) Modification levels of 5gmC at different regions surrounding TE sites. Red dots represent mean values, while white lines indicate median values. Upstream of the TE region is defined by 1 kb regions upstream of the TE start site, and

downstream of the TE is defined by 1 kb regions downstream of the TE end site. Sites with overlapping features from multiple regions were excluded from the statistics.

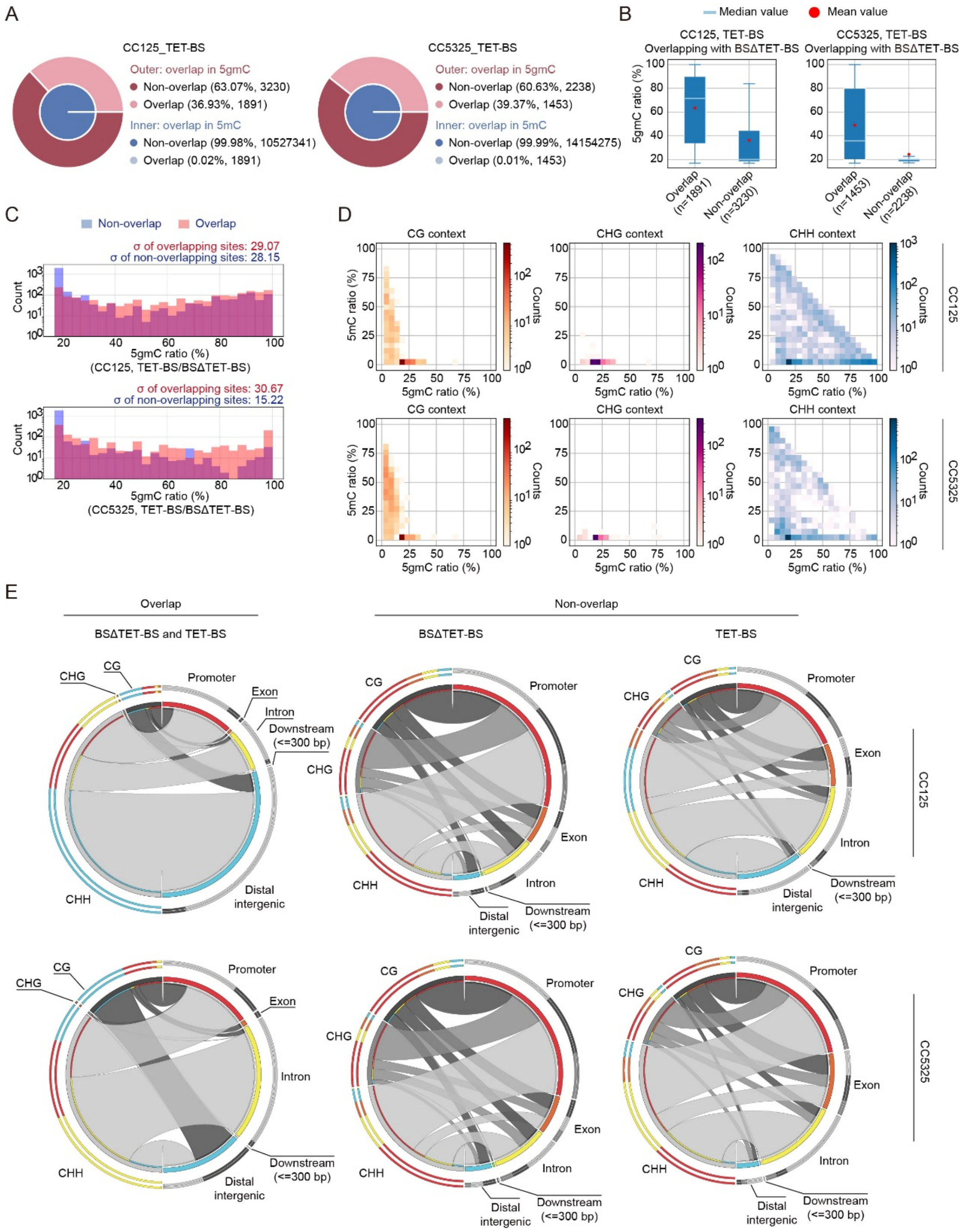

Supplementary Figure S11. Correlation between 5gmC and 5mC determined by TET-BS-seq. (**A**) Overlap analysis of 5gmC and 5mC identified from TET-BS-seq and WGBS. (**B**) Modification levels of 5mC and 5gmC at overlapping and non-overlapping sites. Red dots represent mean values, while white lines indicate median values. The ratios were calculated based on confidently identified 5gmC sites, and the average ratios within these sites were determined. (**C**) Distribution of 5mC and 5gmC counts (y-axis shown on a log10 scale) at overlapping and non-overlapping sites. σ denotes standard deviation. (**D**) Levels of 5mC and 5gmC at confident 5gmC sites across different genomic contexts. (**E**) Site-specific distribution of 5gmC and 5mC in CG, CHG,

and CHH contexts within various functional genomic regions. 5gmC and 5mC at the overlapping and non-overlapping sites are shown separately.

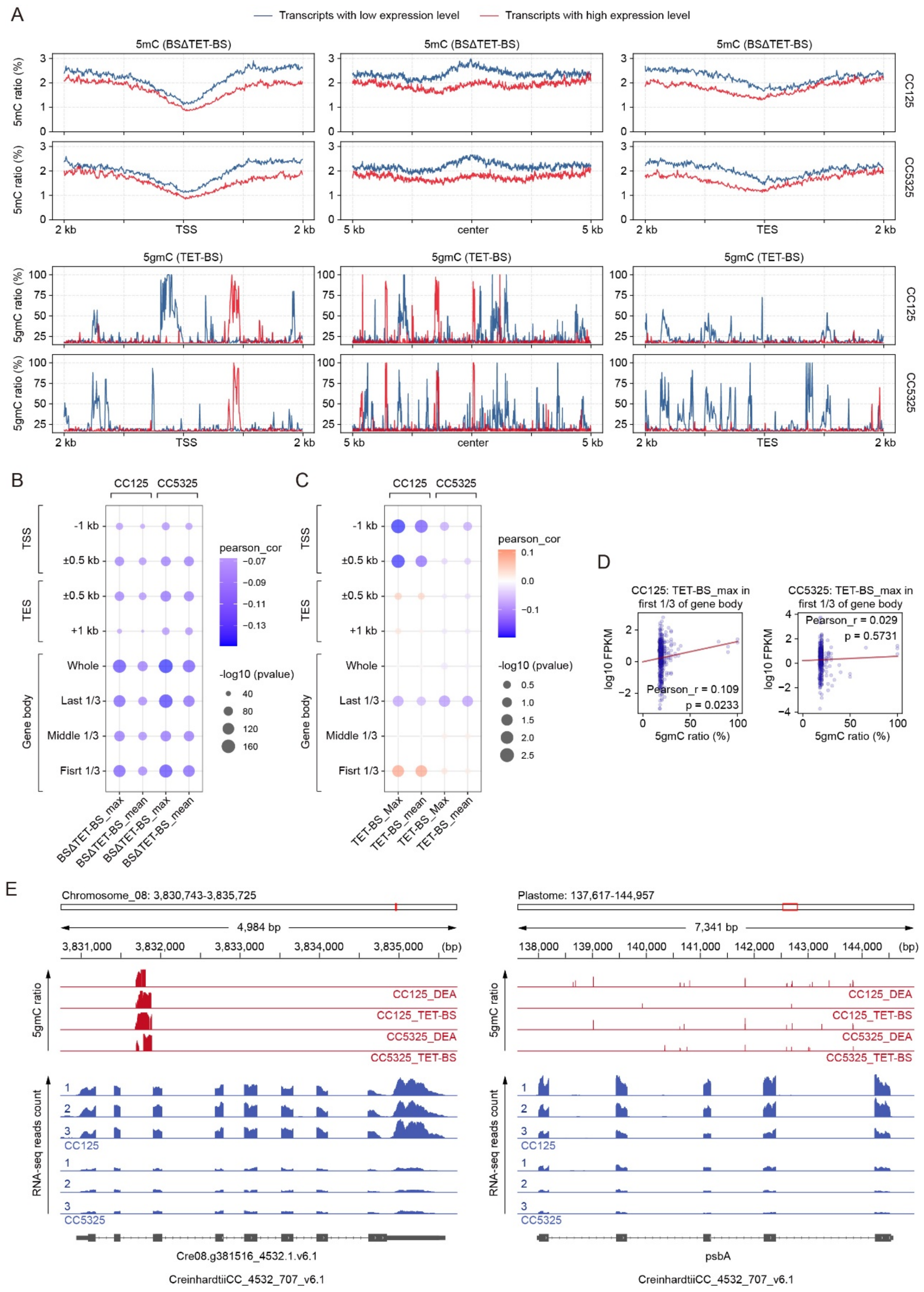

Supplementary Figure S12. Association between 5gmC and gene transcription determined by TET-BS-seq. (**A**) Distribution of 5mC and 5gmC around the TSS, gene body, and TES of differentially transcribed genes. Genes were categorized as highly transcribed (top 25% by FPKM) or lowly transcribed (bottom 25% by FPKM), denoted by red or blue lines, respectively. (**B, C**) Correlation between gene expression and 5mC (**B**) or 5gmC (**C**) levels in different regions as indicated. Modification levels were

characterized using both the maximum and mean values. (**D**) Dot plots showing the positive correlation between transcript levels and 5gmC in the first third of the gene body regions in CC125 and CC5325 cells. (**E**) Representative 5gmC loci in the gene body regions in the genome of *C. reinhardtii*.
